# P_i_ Release Limits the Intrinsic and RNA-Stimulated ATPase Cycles of DEAD-Box Protein 5 (Dbp5)

**DOI:** 10.1016/j.jmb.2015.12.018

**Published:** 2016-01-29

**Authors:** Emily V. Wong, Wenxiang Cao, Judit Vörös, Monique Merchant, Yorgo Modis, David D. Hackney, Ben Montpetit, Enrique M. De La Cruz

**Affiliations:** 1 Department of Molecular Biophysics and Biochemistry, Yale University, New Haven, CT 06520-8114, USA; 2 Department of Cell Biology, University of Alberta, Edmonton, AB, T6G 2H7, Canada; 3 Department of Medicine, University of Cambridge, MRC Laboratory of Molecular Biology, Francis Crick Avenue, Cambridge CB2 0QH, United Kingdom; 4 Department of Biological Sciences and Center for Nucleic Acids Science and Technology, Carnegie Mellon University, Pittsburgh, PA 15213, USA

**Keywords:** DBP, DEAD-box protein, FRET, Förster resonance energy transfer, GCMS, gas chromatography/mass spectrometry, RNA helicase, mRNA export, mantATP, kinetics, thermodynamics

## Abstract

mRNA export from the nucleus depends on the ATPase activity of the DEAD-box protein Dbp5/DDX19. Although Dbp5 has measurable ATPase activity alone, several regulatory factors (e.g., RNA, nucleoporin proteins, and the endogenous small molecule InsP_6_) modulate catalytic activity *in vitro* and *in vivo* to facilitate mRNA export. An analysis of the intrinsic and regulator-activated Dbp5 ATPase cycle is necessary to define how these factors control Dbp5 and mRNA export. Here, we report a kinetic and equilibrium analysis of the *Saccharomyces cerevisiae* Dbp5 ATPase cycle, including the influence of RNA on Dbp5 activity. These data show that ATP binds Dbp5 weakly in rapid equilibrium with a binding affinity (*K*_T_ ~ 4 mM) comparable to the *K*_M_ for steady-state cycling, while ADP binds an order of magnitude more tightly (*K*_D_ ~ 0.4 mM). The overall intrinsic steady-state cycling rate constant (*k*_cat_) is limited by slow, near-irreversible ATP hydrolysis and even slower subsequent phosphate release. RNA increases *k*_cat_ and rate-limiting P_i_ release 20-fold, although P_i_ release continues to limit steady-state cycling in the presence of RNA, in conjunction with RNA binding. Together, this work identifies RNA binding and P_i_ release as important biochemical transitions within the Dbp5 ATPase cycle and provides a framework for investigating the means by which Dbp5 and mRNA export is modulated by regulatory factors.

## Highlights

• mRNA export from the nucleus requires DEAD-box protein Dbp5/DDX19 ATPase activity.
• Kinetics and thermodynamics of intrinsic Dbp5 ATPase reveal RNA's effect on Dbp5.
• Intrinsic Dbp5 ATPase is limited by slow ATP hydrolysis and slower P_i_ release.
• RNA activates P_i_ release, but it and RNA binding still limit RNA-stimulated ATPase.
• RNA binding and P_i_ release define RNA-stimulated Dbp5 ATPase for further regulation.

## Graphical abstract

**Figure:**
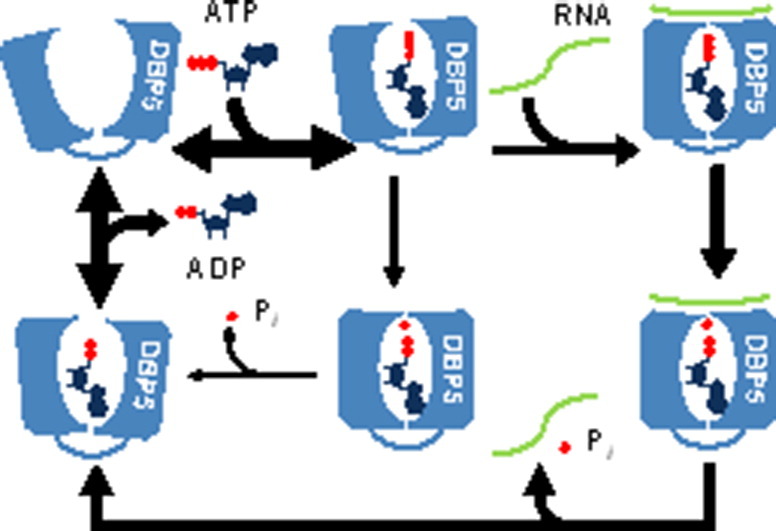

## Introduction

DEAD-box proteins (DBPs) are ATPase enzymes central to RNA metabolism in all domains of life [1], [2], [3]. Many DBPs function as *bona fide* helicases that unwind duplex RNA, while others are involved in RNA–protein complex remodeling [4], [5], [6] and/or RNA folding [7], [8] or serve as RNA sensors within the cell [9], [10]. DBPs accomplish this enormous diversity in function using a highly conserved catalytic core that couples ATP hydrolysis to nucleic acid binding and release [3], [8], [11], [12]. Consequently, DBPs are presumed to perform work via a common minimal enzymatic scheme involving ATP binding, hydrolysis, and subsequent phosphate (P_i_) and ADP product release [8], [11], [13], [14].

The functional diversity of DBPs is thought to stem in part from kinetic and thermodynamic adaptations of this common ATPase cycling scheme [8]. For example, DbpA and Mss116 share a common RNA-activated ATPase cycle pathway, but variations in the catalytic cycle rate and equilibrium constants uniquely alter their interaction with RNA, which may allow them to fulfill specific biological functions [8], [13], [14], [15]. This adaptive behavior is analogous to the duty ratios of cytoskeleton motor proteins and demonstrates how kinetic modulation broadens the biological activities of an enzyme family [8], [16].

The inherent ATPase cycles of some DBPs are additionally regulated through ancillary domains that mediate interactions with regulatory factors that can include RNAs and/or proteins. For example, Dbp5 is a DBP required for nuclear mRNA export that is stimulated by RNA and further regulated by two nucleoporins (Gle1 and Nup159) and an endogenous small molecule (inositol hexakisphosphate or InsP_6_) [17], [18], [19], [20], [21], [22], [23], [24], [25], [26], [27], [28]. While it is clear that all components have global *in vitro* and *in vivo* effects on Dbp5 activity, there is not yet a consensus on the individual and concerted effects of these factors at a mechanistic level. Critical to deciphering the effect of regulators on the ATPase cycle of Dbp5, or any DBP, is a quantitative characterization of the intrinsic ATPase cycle, as it is only with this base knowledge that we can undertake future in-depth studies on the mechanism of regulation by trans-activating factors and apply this to understand the *in vivo* cellular function of DBPs. Deducing how trans-activating factors modulate the ATPase cycle of individual DBPs and the functions that emerge from this regulation will also further our understanding of this important class of enzymes as a whole, which functions in almost all aspects of RNA biology.

Here, we have determined the minimal ATPase cycle and calculated the rate and equilibrium constants of individual biochemical transitions for Dbp5. This work has identified slow ATP hydrolysis and even slower subsequent phosphate release as key bottlenecks in the ATPase cycle of Dbp5 that function as targets for modulation by RNA and other regulatory cofactors. We also show that the fluorescent nucleotides *N*-methylanthraniloyl ATP and ADP (mantATP and mantADP, respectively), common tools for kinetic studies of ATPases [29], [30], [31], [32], [33], significantly alter the ATPase cycle rate and equilibrium constants of Dbp5 (Supplementary Information). Although mant-labeled nucleotides have been found to alter the catalytic cycles of other molecular motors, to our knowledge, this is the first example of such significant mant fluorophore-induced alterations in DBP behavior, and this highlights the ongoing need for system-specific controls regarding fluorescent labels [33], [34], [35], [36].

Having defined the minimal catalytic ATPase cycle of Dbp5, we show that, in the presence of RNA, phosphate release remains a limiting step in the hydrolysis cycle of Dbp5. However, we also observe evidence of a new RNA-binding-associated rate-limiting step in the kinetic scheme of Dbp5, which identifies an additional feature in the minimal kinetic scheme of RNA-stimulated ATPase activity. Overall, by understanding the steps that limit Dbp5 function in various biochemical states (e.g., RNA-bound *versus* free), we are building a framework for understanding Dbp5 functions *in vivo*, which may vary and be dependent upon different regulators given the assorted functions Dbp5 has been proposed to perform [17], [18], [19], [20], [37], [38], [39], [40], [41], [42].

## Results

### Intrinsic ATPase activity of Dbp5

#### Steady-state ATPase measurements

**Fig. 1:**
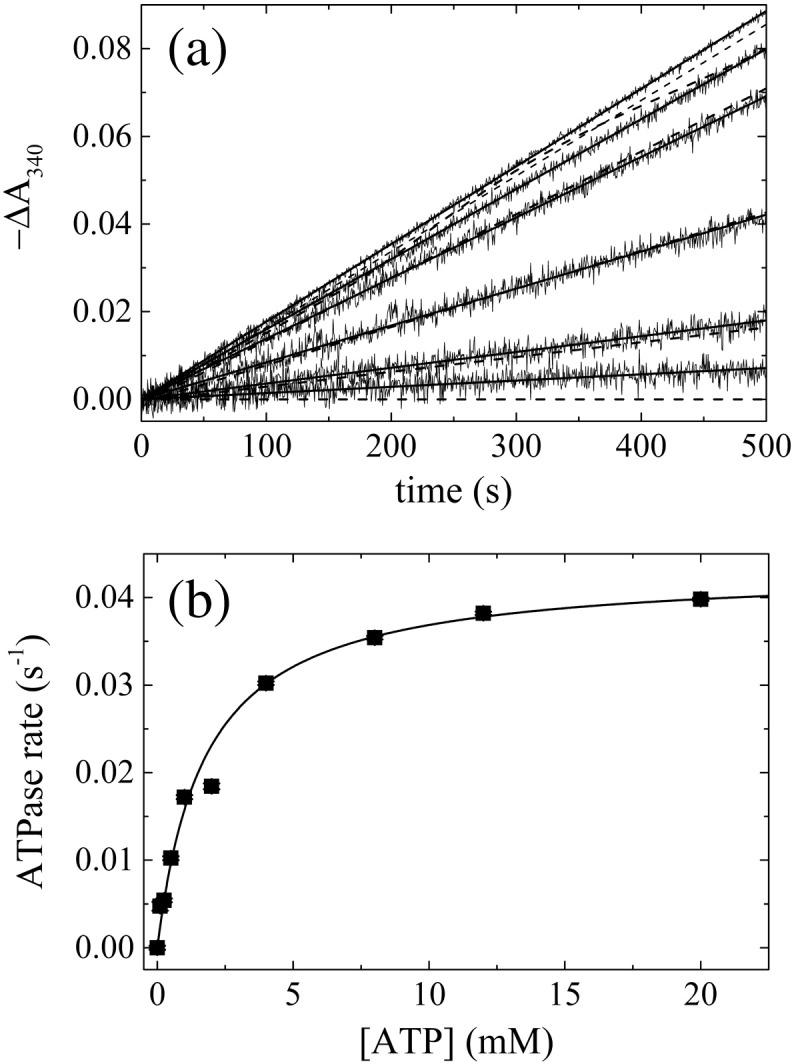
Steady-state ATPase activity of Dbp5. (a) Time courses of absorbance change at 340 nm assayed with the NADH-coupled assay after mixing 660 nM Dbp5 with (lower to upper curves) 0, 0.25, 0.5, 1, 2, 8, or 20 mM ATP. The continuous lines through the data represent the best fits to linear functions, yielding the steady-state ATPase rates from the slopes. Broken lines represent kinetic simulations of Scheme 1 and the corresponding rate and equilibrium constants obtained from global fitting with KinTek Explorer in Table 2. (b) [ATP] dependence of the Dbp5 steady-state ATPase rate. The continuous line through the data represents the best fit to a rectangular hyperbola, yielding the maximum velocity per enzyme (*k*_cat_) from the amplitude and the *K*_M,ATP_ from the [ATP] at half-maximum velocity (Table 1). Uncertainty bars represent standard errors in the fits and are within the data points.

Dbp5 has intrinsic ATPase activity in the absence of RNA or other regulatory factors [18], [20], [22]. As an essential starting point for quantitative analysis of the ATPase cycle of Dbp5, we first determined the maximal per-enzyme cycling rate, or *k*_cat_ (in units of s^− 1^). Under our experimental conditions using both the NADH-coupled assay to detect ADP product formation and P_i_BiP to detect phosphate product accumulation [43], [44], *k*_cat_ is 0.04–0.08 s^− 1^ in the presence of saturating ATP (Fig. 1 and Table 1). The *K*_M_ for ATP substrate (*K*_M,ATP_) is 1.3–1.9 mM (Fig. 1 and Table 1). These values are comparable to published values obtained under different solution conditions [22], [45].

#### Transient kinetic analysis of the Dbp5 ATPase cycle

To define the rate-limiting steps within the intrinsic Dbp5 ATPase cycle, we used a combination of kinetic assays to measure individual rate and equilibrium constants. As described below, binding and dissociation of unlabeled nucleotides (ATP and ADP) were measured by competition with mant-labeled nucleotides, and constants related to ATP hydrolysis and product release were measured with P_i_BiP and oxygen isotope exchange assays.

##### Dbp5 nucleotide binding and dissociation

**Fig. 2:**
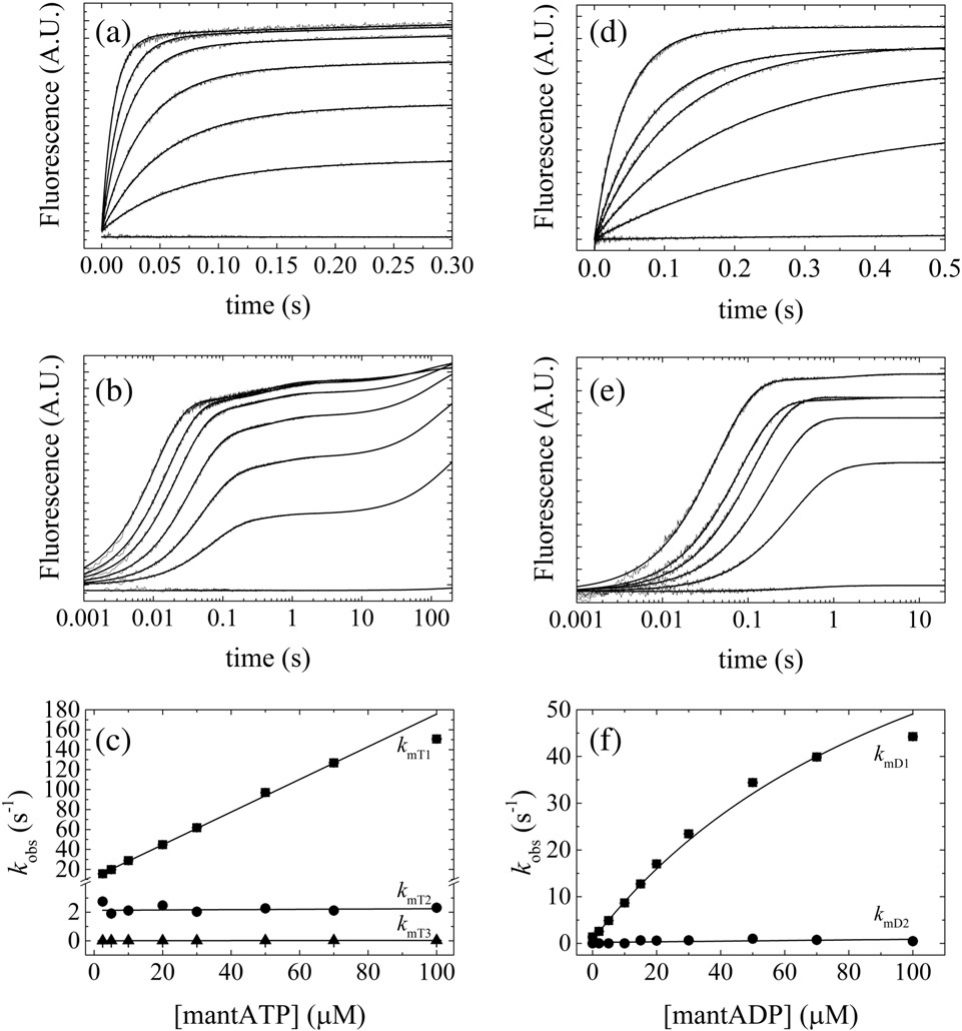
Mant-Nucleotide binding to Dbp5. (a) Time courses of fluorescence change after mixing 0.5 μM Dbp5 with (lower to upper) 0, 2.5, 5, 10, 20, 30, or 50 μM mantATP. The smooth lines through the data represent the best fits to three exponentials. (b) Full time courses from (a) shown on a log scale to 200 s. (c) [mantATP] dependence of the observed rate constants associated with mantATP binding. Continuous lines represent the best linear fits to the data. Uncertainty bars representing the standard errors of the best fits are within the data points. (d–f) Analogous mantADP binding data fitted to two exponentials shown on linear (d) and log (e) scales and with rate constants fitted to hyperbolic or linear functions (f).

The Förster resonance energy transfer (FRET)-based fluorescence enhancement associated with mant-labeled nucleotide binding has been successfully used as a spectroscopic probe to characterize the ATPase cycles of DNA [33], [35], [46], [47], [48], [49] and RNA helicases [13], [14], [30], myosin [50], [51], [52], [53], [54], and kinesin [32], [34], [55], [56] motor proteins, as well as G-proteins [57], [58], [59], with modest perturbations introduced by the mant moiety. We monitored mant-labeled ATP and ADP binding to Dbp5 from changes in FRET between Dbp5 tryptophans and the mant fluorophore. Time courses of fluorescence enhancement after rapidly mixing mantATP with Dbp5 were described by a sum of three exponentials (Fig. 2a and b and Supplementary Information), while mantADP binding was described by a sum of two exponentials (Fig. 2c and d and Supplementary Information). The multi-exponential behavior did not arise from the mixed mant isomers (Supplementary Information) and was therefore modeled as two (mantADP) or three (mantATP) detectable and sequential biochemical transitions. In the case of mantATP, the [mantATP] dependence of the three observed rate constants yields both forward and reverse rate constants (*k*_+ mT*i*_ and *k*_− mT*i*_, *i* = 1, 2, 3; Table 1 and Supplementary Information). The mantATP binding affinity (*K*_mT_; the mantATP equivalent of *K*_T_ in Scheme 1, see Materials and Methods) determined from the ratio of *k*_− mT1_/*k*_+ mT1_ is 7 μM (Table 1).

The *overall* mantATP binding affinity (*K*_mT,overall_, a composite representing mantATP binding and successively populated transitions) is in the range 1 μM (Table 1), several orders of magnitude tighter than the *K*_M_ of unlabeled ATP (*K*_M,ATP_ = 1.3–1.7 mM; Table 1) or the ATP binding affinity (1–2 mM) determined by NMR [45] and this study (discussed below). The > 240-fold difference between mantATP and ATP binding affinities suggests that the mant fluorophore affects nucleotide binding to Dbp5 [33], [35]. To identify the molecular origins of potential perturbations introduced by the mant moiety, we determined the structure of Dbp5 with bound mant nucleotide.

##### RNA/mantADP·BeF_3_-bound Dbp5 is structurally similar to RNA/ADP·BeF_3_-bound Dbp5

The structure of Dbp5 with bound nucleotide has not been determined by X-ray crystallography, but the structure of Dbp5 bound to RNA and the ATP analog (ADP·BeF_3_) has been reported [27]. Therefore, we determined the structure of Dbp5 with bound mantADP·BeF_3_ and RNA to assess the influence of the mant moiety and to identify any structural perturbations in the complex.

**Fig. 3:**
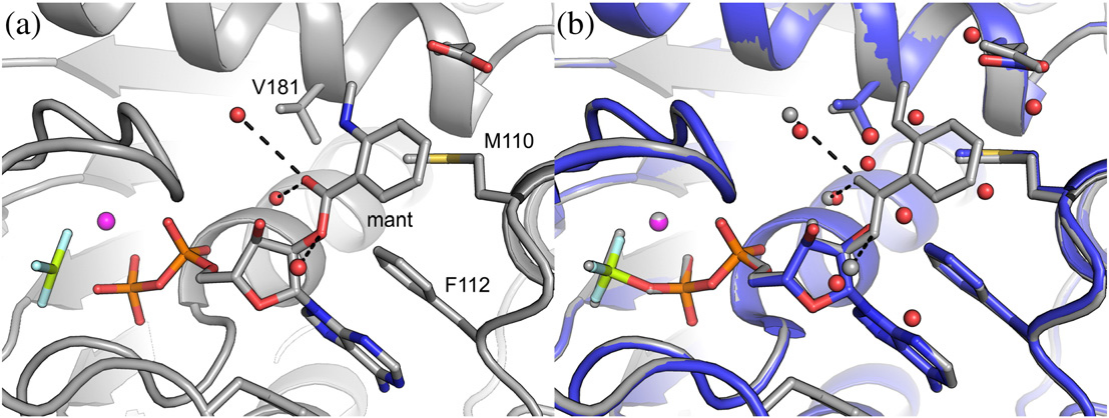
Interactions between mant-ADP·BeF_3_ and Dbp5. (a) The mant fluorophore buries adjacent residues (Val181, M110, and Phe112) of ∆90Dbp5 *via* hydrophobic interactions (< 4.0 Å). The mant moiety hydrogen bonds (broken lines) with waters at the solvent-exposed surface. (b) Displacement of a network of hydrogen-bonded water molecules by the mant fluorophore. ∆90Dbp5-ADP·BeF_3_ (blue) and the associated water molecules (red) of 3PEY superimposed to ∆90Dbp5-mant-ADP·BeF_3_ and the associated water molecules (gray). π Stacking of the adenine is maintained. rmsd = 0.1106 Å.

The crystal structure of RNA/mantADP·BeF_3_-bound Dbp5, refined to a resolution of 1.8 Å, reveals hydrophobic interactions between the mant moiety and three adjacent residues (Met110, Phe112, and Val181; Fig. 3a). Binding of mantADP buries 3397 Å^2^ of the solvent-accessible surface area of Dbp5, with the mant moiety of the ligand accounting for 511 Å^2^. If we assume that such change in hydrophobic surface area provides − 15 ± 1.2 cal mol^− 1^ Å^− 2^ [60], the additional contacts made by the mant fluorophore could potentially provide a total of − 7.6 kcal mol^− 1^ to the binding free energy. The mant moiety would therefore increase the ATP affinity from 6.4 mM (Δ*G*^0′^ = − 3.0 kcal mol^− 1^) to 20 nM (Δ*G*^0′^ = − 10.6 kcal mol^− 1^) and the ADP affinity from 360 μM (Δ*G*^0′^ = − 4.8 kcal mol^− 1^) to 1 nM (Δ*G*^0′^ = − 12.4 kcal mol^− 1^). Although these mant-nucleotide binding constants are considerably tighter than observed (Table 1), it demonstrates that hydrophobic interactions with the mant moiety can account for the tighter binding affinity.

Superposition of RNA/mantADP·BeF_3_-bound Dbp5 onto RNA/ADP·BeF_3_-bound Dbp5 (PDB ID 3PEY) reveals a similar overall structure of the ATPase and bound ligands (rmsd_Cα_ = 0.1106 and rmsd_all-atom_ = 0.3939) [27], suggesting that structural perturbations of Dbp5 and RNA introduced by the mant moiety are minimal (Figs. S4 and S5). One notable difference between the superimposed structures is the displacement of a set of eight water molecules found in the nucleotide-binding site of the ADP·BeF_3_-bound structure by the mant moiety (Fig. 3b), which may further contribute to the differences in mant-nucleotide binding.

##### Unlabeled nucleotide binding to Dbp5

**Fig. 4:**
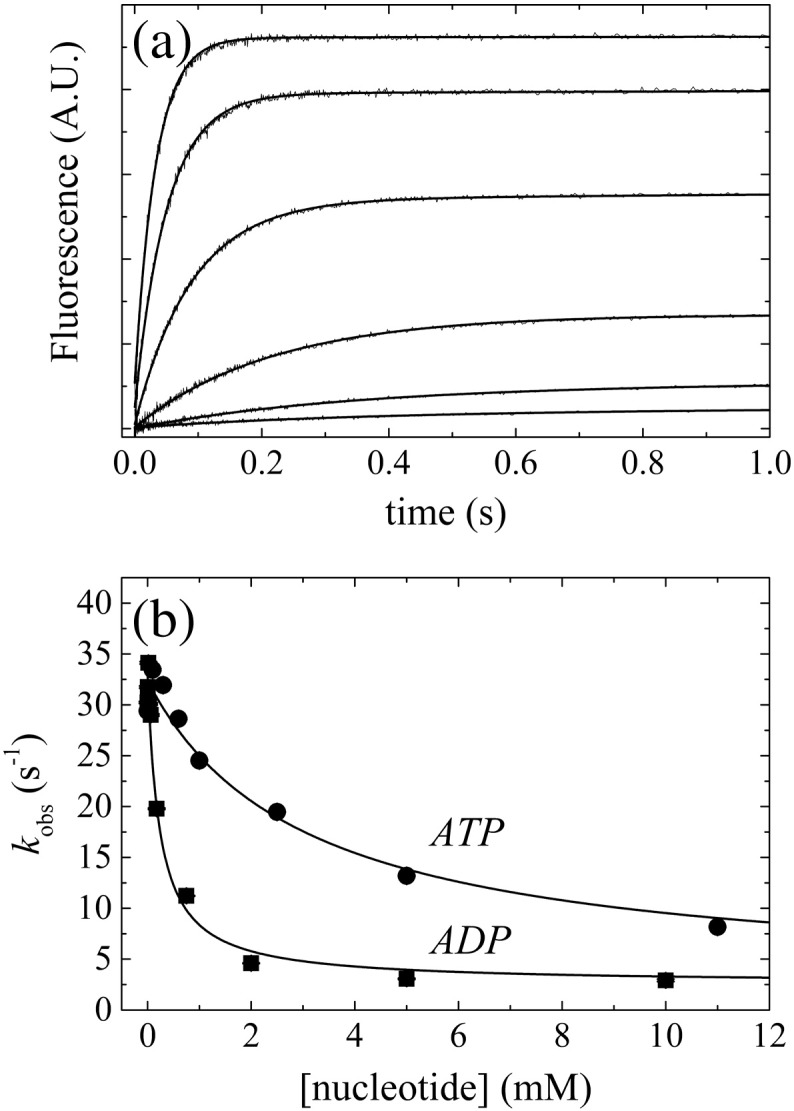
Unlabeled nucleotide binding affinity measured by competition with mant nucleotides. (a) Time courses of fluorescence change after mixing 0.5 μM Dbp5 with 50 μM mantADP and 0, 0.175, 0.75, 2, 5, or 10 mM ADP (upper to lower). Continuous lines through the data represent best fits to two exponentials. (b) [nucleotide] Dependence of the fastest observed rate constant (*k*_1,obs_) after mixing 0.5 μM Dbp5 with a solution of 50 μM mantADP supplemented with 0, 0.1, 0.3, 0.6, 1, 2.5, 5, or 11 mM ATP (black squares). Black circles represent the fastest observed rate constant of mantADP binding on mixing 0.5 μM Dbp5 with a solution of 50 μM mantADP supplemented with 0.005, 0.015, 0.03, 0.0625, 0.175, 0.75, 2, 5, or 10 mM ADP. Continuous lines represent best fits to Eq. (13).

Kinetic competition between mantATP or mantADP and unlabeled ATP was performed to obtain parameters for unlabeled ATP binding and dissociation. ATP slows all observed rate constants associated with mantATP and mantADP binding (Fig. 4 and Table 1), consistent with ATP binding in a rapid equilibrium (> 300 s^− 1^) and competing with mant nucleotides for Dbp5 binding [51], [61]. Rapid ATP binding kinetics are beyond stopped-flow detection limits (~ 2 ms); thus, we used the reduction in *k*_mT1,obs_ with increasing [ATP] to estimate an unlabeled ATP binding affinity (*K*_T_) of 6 mM [Fig. 4b, Eq. (13), and Table 1]. Competition with mantADP yields an ATP affinity of 3 mM (Fig. 4 and Table 1). The comparable (i.e., < 2-fold difference) ATP binding affinities determined from competition with mantATP or mantADP suggests that mantATP turnover (i.e., hydrolysis, product release, and any steady-state ATPase activity) occurs much more slowly than initial binding of ATP and does not affect the measured *K*_T_ (see Supplementary Information).

Kinetic competition between mantADP and ADP demonstrates that Dbp5 binds ADP in rapid equilibrium but ~ 10-fold more tightly than ATP (*K*_D_ = 360 μM; Table 1 and Fig. 4), in agreement with published values (400–570 μM) [45]. Thus, mant-labeled nucleotides bind Dbp5 more tightly than their unlabeled counterparts (Table 1). The multiple observed phases in mant-nucleotide binding, particularly those of mantADP binding, may reflect isomerization steps facilitated by additional interactions between Dbp5 and the fluorophore. These isomerizations may also contribute to high-affinity binding of mant-labeled nucleotides.

##### Dbp5 ATP hydrolysis and phosphate release

**Fig. 5:**
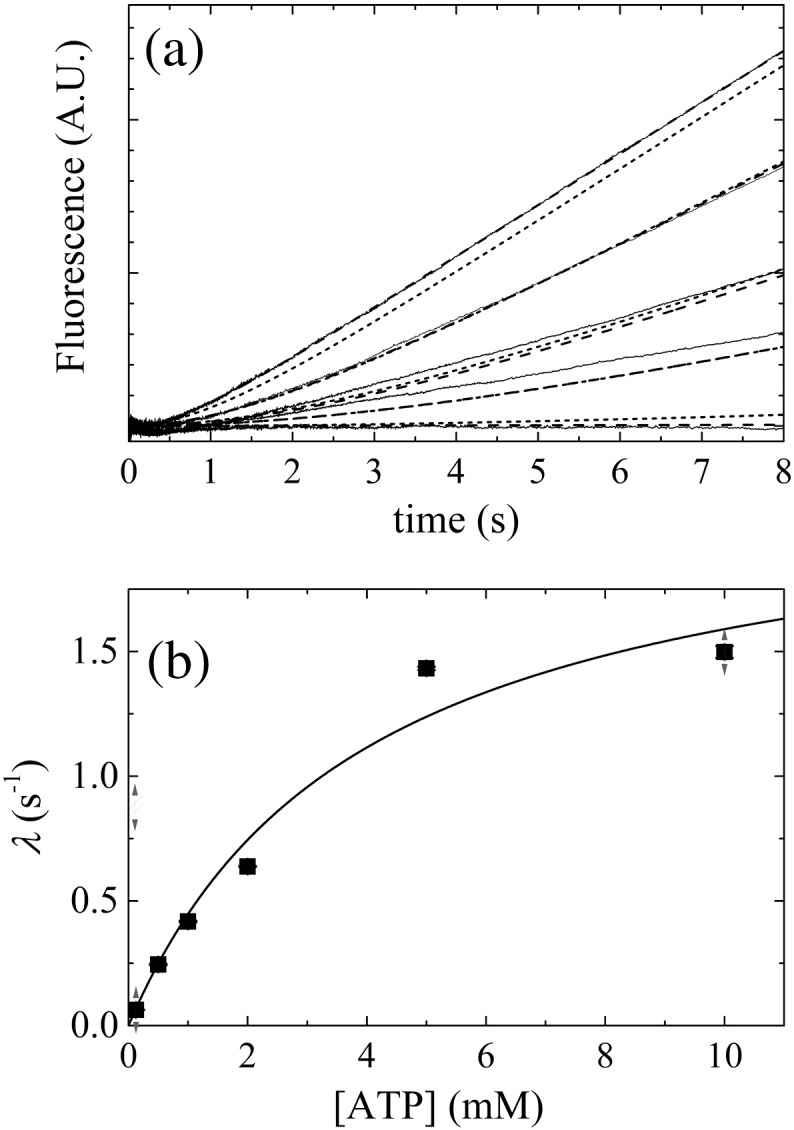
Transient and steady-state P_i_ release during Dbp5 ATPase cycling. (a) Time courses of fluorescence change after mixing 0.5 μM Dbp5 with 5 μM P_i_BiP containing (lower to upper curves) 0, 0.5, 1, 2, or 5 mM ATP. Fits to Eq. (14) are shown as broken lines through the data. Simulated data (using rate and equilibrium constants obtained from KinTek Explorer global fitting; Table 2) are shown as dotted lines through the data. (b) [ATP] dependence of the observed P_i_ release lag phase rate constant (λ). Continuous line through the data points represents best fit to a rectangular hyperbola [Eq. (15)].

Fluorescently labeled phosphate binding protein (P_i_BiP) has been used to measure transient P_i_ release from DBPs and other molecular motor proteins [13], [14], [44], [53]. Its tight binding of P_i_ (*K*_d_ = 0.1 μM) and rapid association rate constant (> 3 × 10^8^ M^− 1^ s^− 1^) provides real-time fluorescent detection of transient and steady-state P_i_ release [62]. Time courses of P_i_BiP fluorescence enhancement after rapidly mixing Dbp5 with ATP display multiple phases. The most prominent features are a lag followed by a linear phase (Fig. 5). The linear portion corresponds to steady-state ATPase cycling. The steady-state cycling parameters (*k*_cat_ = 0.08 s^− 1^ and *K*_M,ATP_ = 1.3 mM) obtained from fitting the [ATP] dependence of the turnover rate (i.e., the slope of the linear regime) to Eq. (10) agree well with those obtained with the NADH-coupled assay (Table 1), providing independent determination of the steady-state parameters (Table 1).

The lag phase is defined by an observed rate constant whose value depends hyperbolically on the [ATP], yielding an ATP binding affinity (*K*_T_) of 4 mM [Fig. 5 and Eq. (15)], comparable to the values obtained by competition with mantATP and mantADP (Fig. 4). The observed lag phase rate constant at saturating ATP (λ_∞_) has a value of 2 s^− 1^ and corresponds to the sum of the ATP hydrolysis and P_i_ release rate constants (Scheme 1) as follows[13], [14]:

(1) $$\lambda_{\infty }=k_{+H}+k_{-H}+k_{-P_{i}}$$

The intercept of the lag phase at zero ATP (λ_0_) is defined by:

(2) $$\begin{array}{l} \lambda_{0}=\frac{k_{-T}k_{-H}+k_{-T}k_{-P_{i}}+k_{+H}k_{-P_{i}}}{k_{-T}}\\ \;=k_{-H}+k_{-P_{i}}+\frac{k_{+H}k_{-P_{i}}}{k_{-T}} \end{array}$$

and has a value of ~ 0.02 s^− 1^, though this is subject to experimental uncertainty since it is close to the origin. When ATP dissociation is rapid (*k*_− T_ ≫ other rate constants preceding ADP release in Scheme 1, a condition that is fulfilled since ATP binds in rapid equilibrium), the net contributions from the third term (*k*_+ H_*k*_− Pi_/*k*_− T_) of Eq. (2) are negligible and λ_0_ simplifies to the sum of ATP resynthesis (*k*_− H_) and P_i_ release (*k*_− Pi_) rate constants:

(3) $$\lambda_{0}∼k_{-H}+k_{-P_{i}}$$

Combining Eqs. (1), (3) allows the observed lag phase rate constant to be expressed as:

(4) $$\lambda_{\infty }∼k_{+H}+\lambda_{0}$$

Given the values of λ_∞_ (~ 2 s^− 1^) and λ_0_ (~ 0.02 s^− 1^), we conclude that ATP hydrolysis (*k*_+ H_) dominates λ_∞_ and occurs with a rate constant of ~ 2 s^− 1^ (Table 1). This and a steady-state cycling *k*_cat_ of 0.08 s^− 1^ indicate that ATP hydrolysis is not rate limiting, though it can contribute to the value of *k*_cat_[13], [14], [15].

##### Oxygen isotope exchange

Oxygen isotopic exchange experiments were performed to determine the ratio between reverse hydrolysis (*k*_− H_) and phosphate release (*k*_− Pi_). After hydrolysis, Dbp5 with bound ADP-P_i_ will either release P_i_ or resynthesize ATP, depending on the relative rate constants for P_i_ release and reverse hydrolysis. In each ATP hydrolysis event, a water-derived oxygen is incorporated into the liberated P_i_ and there is a defined probability (depending on the solution enrichment of ^18^O) that the water-derived oxygen is a heavy isotope. Therefore, the ^18^O isotope content in the released P_i_ product reveals the number of hydrolysis events that occurred before P_i_ release and determine the probability ratio of ATP resynthesis *versus* phosphate release [13], [15], [61], [63]. In the absence of significant reverse hydrolysis, P_i_ will have a single water-derived oxygen, but significant reverse hydrolysis will generate P_i_ with multiple water-derived oxygens due to multiple rounds of hydrolysis and resynthesis prior to P_i_ release.

The *P*_c_ value is determined by measuring the relative populations of singly and multiply labeled ^18^O phosphates and is defined by the ATP resynthesis and P_i_ release constants according to the following[61], [63], [64]:

(5) $$P_{c}=\frac{k_{-H}}{k_{-H}+k_{-P_{i}}}$$

The ratio of the ^18^O_2_:^18^O_1_ species was 0.0101 ± 0.0009 for hydrolysis by Dbp5 in 48.5% ^18^O water (see Materials and Methods for full description of data analysis). This ratio corresponds to a *P*_c_ value of 0.0113 ± 0.0026, indicating that P_i_ release occurs almost 100-fold faster than reverse hydrolysis (*k*_− Pi_/*k*_− H_ = 99) and little ATP resynthesis occurs during steady-state cycling. Consequently, the *y*-intercept of the lag phase rate constant λ_0_[Eq. (2)] is dominated by *k*_− Pi_ with a value of ~ 0.02 s^− 1^ and a *k*_− H_ value of 0.0002 s^− 1^[Eq. (3) and Table 1]. Thus, Dbp5 hydrolysis is essentially irreversible despite slow P_i_ release. The determined value of the phosphate release rate constant of *k*_− Pi_ ~ 0.02 s^− 1^ is comparable to the steady-state cycling rate, suggesting that it or a transition preceding rapid P_i_ release limits Dbp5 cycling.

##### P_i_ rebinding during ATPase cycling

To assess if P_i_ rebinding contributes to overall Dbp5 ATPase cycling and to estimate the P_i_ rebinding rate constant, we used the NADH assay (P_i_ release is irreversible in the P_i_BiP assay due to P_i_BiP and mop) to measure Dbp5 steady-state ATPase activity over a wide range of added free P_i_. The Dbp5 ATPase activity is not affected by inclusion of P_i_ up to 10 mM, yielding an apparent $K_{P_{i},SS}=\frac{\left[P_{i}\right]\left[HD\right]}{\left[{HDP}_{i}\right]}$ value > 10 mM during steady-state cycling and making P_i_ release essentially irreversible.

Although the steady-state distributions of the biochemical states are not identical with the equilibrium distributions, it is possible to relate this apparent *K*_Pi,SS_ to the true equilibrium constant ($K_{P_{i},\mathrm{eq}}=\frac{k_{-P_{i}}}{k_{+P_{i}}}$) with consideration of reaction flux. During initial steady-state cycling, the reaction flux at every transition (Scheme 1) is constant and equal to the observed product release rate, *v*_0_[Eq. (10)]. For P_i_ release and rebinding, this is given by the expression

(6) $$k_{-P_{i}}\left[{HDP}_{i}\right]-k_{+P_{i}}\left[P_{i}\right]\left[HD\right]=v_{0}\leq {\left[H\right]}_{T}k_{\mathrm{cat}}$$

Dividing both sides of Eq. (6) by *k*_+ Pi_[HDP_i_], rearranging terms, and substituting in the steady-state and equilibrium definitions of *K*_Pi_ yields

(7) $$k_{P_{i},\mathrm{eq}}=\frac{k_{-P_{i}}}{k_{+P_{i}}}=\frac{\left[P_{i}\right]\left[HD\right]}{\left[{HDP}_{i}\right]}+\frac{v_{0}}{k_{+P_{i}}\left[{HDP}_{i}\right]}=K_{P_{i},SS}+\frac{v_{0}}{k_{+P_{i}}\left[{HDP}_{i}\right]}>K_{P_{i},SS}$$

indicating that the affinity of P_i_ at equilibrium is always weaker than that in steady state. Therefore, since *K*_Pi,eq_ > 10 mM and *k*_− Pi_ ~ 0.02 s^− 1^, *k*_+ Pi_ must be slower than 2 × 10^− 6^ s^− 1^. These data suggest that P_i_ rebinding by Dbp5 is negligible under our assay conditions and does not result in substantial product inhibition, even in the absence of the P_i_ mop.

#### Kinetic simulations from global fitting

Our transient kinetic analysis indicates that the intrinsic ATPase cycle of Dbp5 features rapid, weak ATP binding, moderately slow hydrolysis, and rate-limiting P_i_ release followed by rapid ADP dissociation. To verify the experimentally determined rate and equilibrium constants, we globally fit ATPase time courses acquired with NADH and P_i_BiP assays (Fig. 1, Fig. 5) at different [Dbp5] and [ATP] to Scheme 1 using KinTek Explorer software. All the rate constants in Scheme 1 were unconstrained in global fitting with the exception of the following ratios: the ATP and ADP binding affinities (defined by the ratio of *k*_−_/*k*_+_) were constrained to the experimentally determined values of 6 mM and 310 μM, respectively, and P_i_ release was constrained to a value ~ 100-fold faster than reverse hydrolysis. Our only fully constrained rate constant was P_i_ rebinding, which was set at a near-zero value since P_i_ release from Dbp5 is essentially irreversible during steady-state cycling (and is irreversible in the presence of P_i_BiP). The fundamental rate constants obtained from global fitting (Table 2) agree well with the experimentally determined rate constants (Table 1) and account for the experimental time courses for various assays.

Global fits by KinTek Explorer closely match the experimental NADH assay data (Fig. 1) when P_i_ release is either partially or solely rate limiting. The requirement for two partially rate-limiting steps (from the distinguishing lag phase in transient phosphate release assays; Fig. 5) eliminates scenarios in which hydrolysis does not also contribute to *k*_cat_. In support of this scheme, simulations generated from global fitting outputs are very sensitive to changes in both ATP hydrolysis/resynthesis and P_i_ release but are insensitive to changes in ATP/ADP binding and dissociation provided that those steps are much faster than hydrolysis and P_i_ release. The best global fit of the NADH experimental data was achieved with ATP hydrolysis (*k*_+ H_) at 0.16 s^− 1^, ATP resynthesis (*k*_− H_) at 6 × 10^− 5^ s^− 1^, and P_i_ release (*k*_− Pi_) at 0.06 s^− 1^ with minimal phosphate rebinding (*k*_+ Pi_ ~ 0), thereby making P_i_ release the major determinant of *k*_cat_ (Table 2).

We also globally fitted transient P_i_ release data (Fig. 5) with thermodynamically constrained rapid ATP/ADP binding and dissociation and a *P*_c_ value of 0.01. Independent of the steady-state NADH assay global fits by KinTek Explorer, the transient P_i_ release time courses are best globally fitted by a mechanism in which P_i_ release is the major determinant of *k*_cat_: ATP hydrolysis (*k*_+ H_) occurs at 0.62 s^− 1^, ATP resynthesis (*k*_− H_) occurs at 0.001 s^− 1^, and P_i_ release (*k*_− Pi_) occurs at 0.1 s^− 1^. Constraining ATP hydrolysis (*k*_+ H_) to 0.16 s^− 1^ (the best global fit output from the NADH assay data) yields simulation outputs that overlay with the P_i_BiP data (Table 2 and Fig. 5). Once again, in all converged parameters, P_i_ release limits the *k*_cat_ of Dbp5.

The steady-state cycling parameters *k*_cat_ and *K*_M,ATP_ can also be calculated from fundamental rate constants, but the relationship depends on the kinetic scheme. Using a minimal four-state model (Scheme 1), making no assumptions about the speed or reversibility of any of the associated transitions, the theoretical *k*_cat_ and *K*_M,ATP_ values are given by[13], [65]:

(8) $$k_{\mathrm{cat}}=\frac{k_{+H}k_{-P_{i}}k_{-D}}{\left(k_{+H}+k_{-H}\right)\left(k_{+P_{i}}+k_{-D}\right)+k_{+H}k_{-P_{i}}+k_{-P_{i}}k_{-D}}$$

(9) $$K_{M,ATP}=\frac{k_{-T}\left(k_{-H}\left(k_{+P_{i}}+k_{-D}\right)+k_{+P_{i}}k_{-D}\right)+k_{+H}k_{-P_{i}}k_{-D}}{k_{+T}\left(\left(k_{+H}+k_{-H}\right)\left(k_{+P_{i}}+k_{-D}\right)+k_{+H}k_{-P_{i}}+k_{-P_{i}}k_{-D}\right)}$$

Substituting the rate constants derived from global fitting of either steady-state NADH or P_i_BiP assays (Table 2) into the abovementioned equations yields a *k*_cat_ of 0.04 s^− 1^ and a *K*_M,ATP_ of 1.1 mM, which closely match our experimental steady-state data (0.04–0.08 s^− 1^ and 1.3–1.5 mM, respectively; Table 1).

In summary, the rate constants determined from globally fitting our experimental data to Scheme 1 (Table 2) are consistent with those determined experimentally (Table 1). Moreover, the global enzyme cycling parameters, *k*_cat_ and *K*_M_, calculated using individual rate constants obtained from global fitting are comparable to the values determined experimentally. The high degree of agreement between experimentally derived parameters (both steady-state ATPase and transient kinetics) and global fitting outputs demonstrates the robustness of the proposed kinetic scheme and the experimentally determined parameters. As such, the minimal ATPase scheme used here to model Dbp5 activity is sufficient to account for all observed transitions, and it serves as a quantitative framework for evaluating the influence of RNA and regulatory factors.

### Alteration of the intrinsic Dbp5 ATPase cycle by RNA

#### P_i_ release in the presence of RNA

**Fig. 6:**
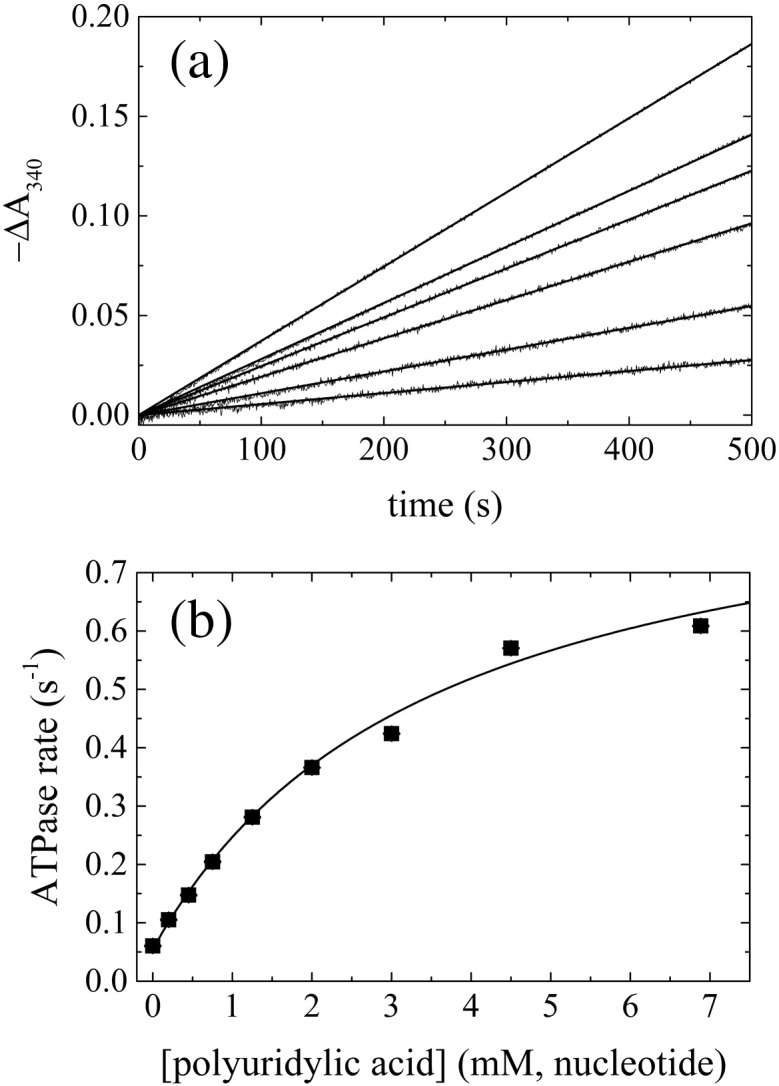
RNA-stimulated steady-state ATPase activity of Dbp5. Time courses of absorbance change at 340 nm assayed with the NADH-coupled assay after mixing 100 nM Dbp5 and 20 mM ATP with (lower to upper curves) 0, 0.45, 1.25, 2, 3, or 4.5 mM polyuridylic acid (concentration refers to total nucleotides). The continuous lines through the data represent the best fits to linear functions, yielding the steady-state ATPase rates from the slopes. (b) [ATP] dependence of the RNA-stimulated Dbp5 steady-state ATPase rate. The continuous line through the data represents the best fit to Eq. (11), yielding the maximum velocity per enzyme (*k*_cat_) from the amplitude and the *K*_M,RNA_ from the [ATP] at half-maximum velocity (Table 1). Uncertainty bars represent standard errors in the fits and are within the data points.

RNA significantly impacts the Dbp5 ATPase cycle [22]. To quantify these changes within the framework described thus far, we performed steady-state ATPase assays with Dbp5 in the presence of saturating levels of ATP (20 mM) and varying [RNA] (up to 7 mM), which resulted in an ~ 20-fold acceleration of *k*_cat_ (0.92 ± 0.08 s^− 1^), confirming earlier studies (Fig. 6 and Table 1) [22]. Given that intrinsic Dbp5 ATPase cycling is mainly limited by P_i_ release, RNA must minimally accelerate P_i_ release 20-fold to achieve this increase in *k*_cat_. We therefore evaluated if RNA had the predicted effect on P_i_ release and also whether or not our modeled rate constants from Scheme 1 were predictive of overall intrinsic Dbp5 steady-state cycling.

**Fig. 7:**
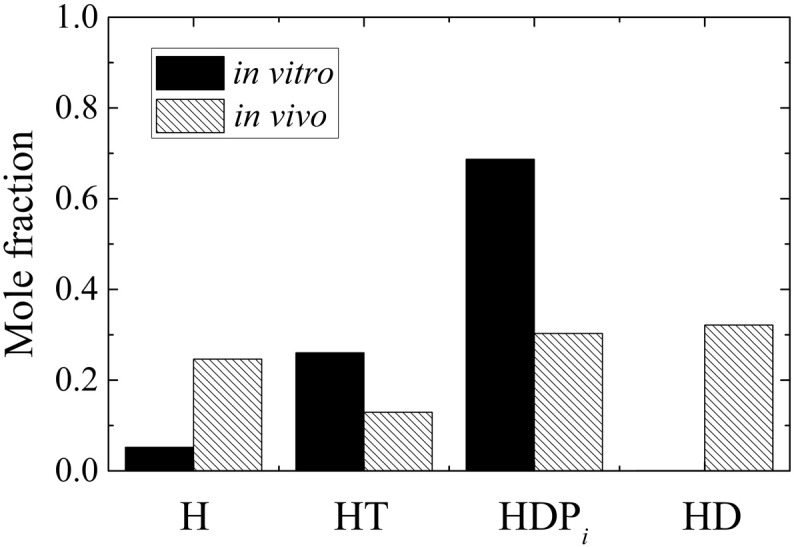
Steady-state distribution of Dbp5 ATPase cycle intermediates. *In vitro* conditions are 20 mM ATP, 0 mM ADP, and 0 mM P_i_. *In vivo* conditions are 2.1 mM ATP [72], 470 μM ADP [72], 2.5 mM P_i_[73].

P_i_ release is primarily rate limiting in the absence of RNA and other cofactors. Therefore, the majority of Dbp5 populates the ADP-P_i_ state under *in vitro* steady-state cycling conditions (Fig. 7). We took advantage of this Dbp5-ADP-P_i_ accumulation in a series of dual-mixing experiments to test the effects of RNA on P_i_ release from the pre-formed Dbp5-ADP-P_i_ state. Dbp5 was first rapidly mixed with ATP and “aged” for various times to allow biochemical intermediates along the ATPase cycle to form, then rapidly mixed again with a range of [RNA] while monitoring P_i_ release with P_i_BiP. Based on the intrinsic rates determined above, delay times of 0.05 s, 6 s, and 25 s should result in progressive Dbp5 population of ATP-bound (pre-hydrolysis), ADP-P_i_ -bound (post-hydrolysis), and ADP-bound (post P_i_ release) states, respectively.

**Fig. 8:**
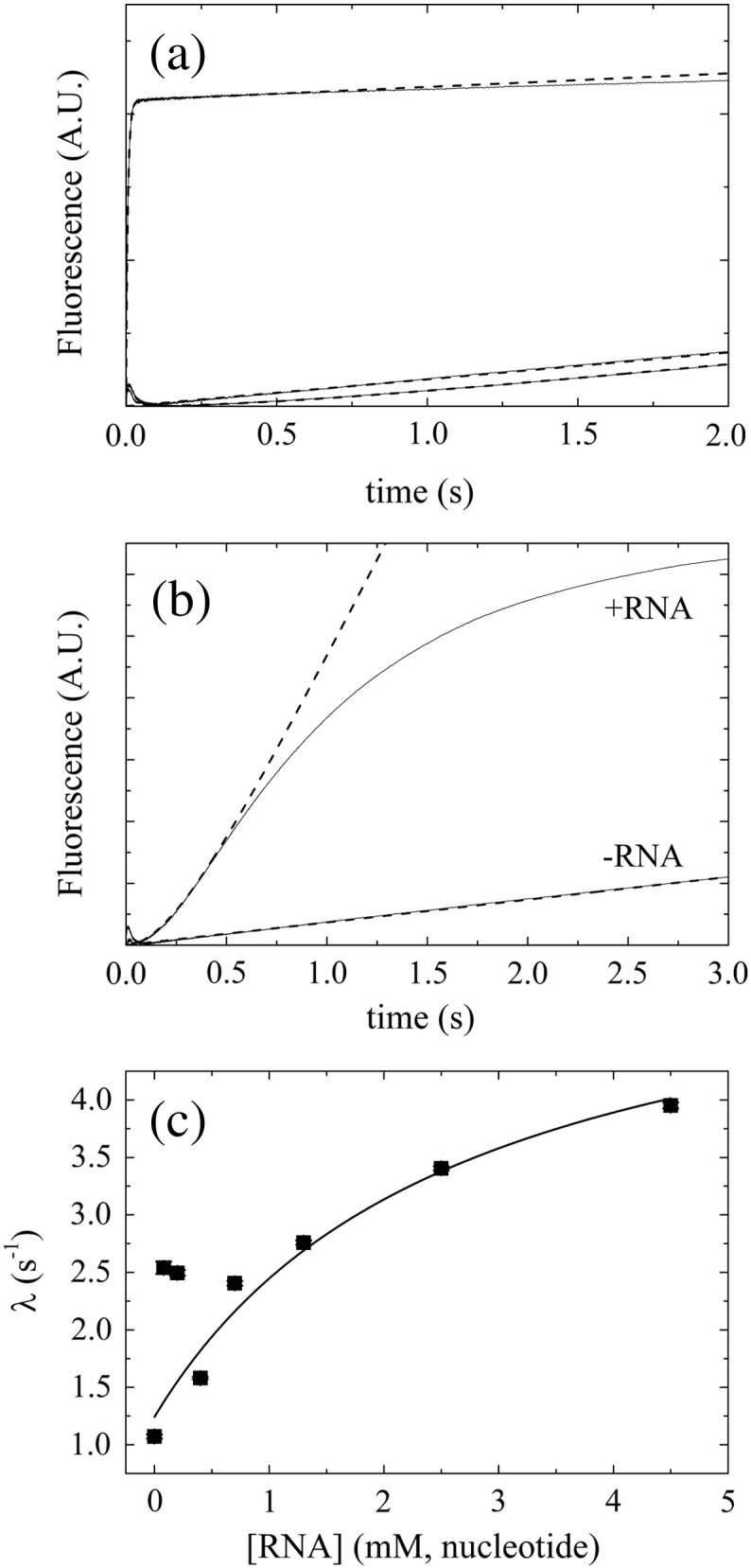
Transient RNA-stimulated P_i_ release during Dbp5 ATPase cycling. (a) Time courses of fluorescence change after mixing 5 μM Dbp5 with 4 mM ATP and aging for (lower to upper) 0.05, 6, or 25 s prior to rapidly mixing with 5 μM P_i_BiP. Broken lines are best fits to (lower to upper) Eq. (14), linear equation, or bimolecular binding plus a linear phase, respectively. (b) Time courses of fluorescence change after mixing 5 μM Dbp5 with 4 mM ATP, aging for 6 s, and then rapidly mixing with 0 or 10 mM RNA in the presence of 5 μM P_i_BiP. Fits to Eq. (16) are shown as broken lines through the data. (c) [RNA] dependence of the observed P_i_ release lag phase rate constant (λ) where pre-equilibrated Dbp5 and RNA are rapidly mixed with ATP and P_i_BiP. Continuous line through the data points represents best fit to a rectangular hyperbola [Eq. (15)], yielding a maximum observed rate constant of 5.5 ± 2 s^− 1^ upon extrapolation of the fit to saturating RNA. The [RNA] at half maximal is ~ 3 mM under these nonsaturating ATP concentrations.

Delay times ≥ 6 s abolished the lag phase in P_i_BiP fluorescence time courses with Dbp5 alone (i.e., no RNA; Fig. 8a). This observation is consistent with formation of the post-hydrolysis ADP-P_i_ state during aging, thereby bypassing (at least) one of the transitions contributing to the lag phase observed in single-mixing experiments (Fig. 5). Furthermore, a rapid burst of P_i_BiP fluorescence was observed with longer delay times (25 s; Fig. 8a), consistent with P_i_BiP binding to P_i_ liberated by Dbp5 via hydrolysis events that occurred during aging. From these data, we can conclude that our modeled rate constants for intrinsic Dbp5 cycling are predictive of actual Dbp5 behavior and that these dual-mixing experiments can be used to interrogate the effect of RNA on P_i_ release.

Upon addition of RNA in the second mix after aging, time courses of P_i_BiP fluorescence display a lag phase followed by a linear phase (Fig. 8b), independent of the age time (from 0 to 25 s) even if the lag is absent from time courses with Dbp5 alone (e.g., 6 s age time). The lag phase observed rate constant with 10 mM RNA is ~ 4–6 s^− 1^ for all aging periods examined. The lag phase observed rate constant also depends hyperbolically on [RNA], reaching a maximum value of ~ 5.5 (± 2) s^− 1^ at saturating [RNA] (Fig. 8c). Thus, RNA alters the hydrolysis cycle of Dbp5 by introducing two or more new rate-limiting steps, at least one of which does not depend on pre-population of a particular Dbp5-nucleotide-bound state. This is most likely an RNA-binding-dependent transition—either slow RNA binding or a slow isomerization following binding—which must occur prior to establishing a new steady state.

The [RNA] dependence of the lag (Fig. 8c) is presumably related to the RNA affinity (*K*_d,RNA_) of Dbp5 (with bound nucleotide) at sub-saturating [ATP], and its value of 3 (± 3) mM agrees well with the Michaelis constant *K*_M,RNA_[14] of 3.4 (± 0.8) mM for [RNA]-dependent Dbp5 ATPase obtained from steady-state NADH assays (Fig. 6b and Table 1). The lack of a P_i_ burst from the pre-formed Dbp5-ADP-P_i_ state following RNA binding (delay times ≥ 6 s; Fig. 8b) also establishes that P_i_ release must continue to be slow and partially rate limiting, even when RNA is present. However, we note that the dual-mixing experiments do not rule out the possibility that hydrolysis may also limit *k*_cat_ in the presence of RNA. We therefore conclude that at least two biochemical transitions—an isomerization associated with RNA binding and P_i_ release—limit RNA-activated Dbp5 ATPase cycling.

#### Oxygen isotope exchange in the presence of RNA

RNA may also promote ATP hydrolysis by Dbp5, as with other DBPs [8], [66], [67]. A faster observed hydrolysis rate constant could result from accelerated ATP resynthesis, ATP hydrolysis, or both. The very slow rate of ATP resynthesis in the intrinsic Dbp5 ATPase cycle makes the first option a distinct possibility, and this is crucial to understanding the potential effects of other regulators. We therefore performed oxygen isotope exchange analysis of the RNA-activated, steady-state Dbp5 ATPase.

The *P*_c_ value in the presence of RNA is 0.042 ± 0.003 (*versus* 0.01 in the absence of RNA), which equates to a P_i_ release rate constant that is ~ 25-fold faster than ATP resynthesis (Table 1). Because P_i_ release is accelerated 20-fold in the presence of RNA, ATP resynthesis must also be accelerated to maintain the observed 4-fold difference in *P*_c_ value. Using the rate constants for P_i_ release, the *P*_c_values, and Eq. (5), we estimate that RNA accelerates ATP resynthesis by Dbp5 ~ 80-fold. Accelerating ATP resynthesis increases the observed ATP hydrolysis rate constant (i.e., shortens ATP hydrolysis relaxation time), as it is determined by the sum of forward and reverse rate constants [68].

## Discussion

### General features of the intrinsic Dbp5 ATPase cycle

Dbp5 has a slow maximum turnover rate (*k*_cat_: 0.04 s^− 1^) and a weak *K*_M,ATP_ (1.3–1.9 mM). The slow *k*_cat_ results from slow ATP hydrolysis coupled to even slower P_i_ release, both of which limit overall cycling, though P_i_ release is the major determinant. It is experimentally difficult to measure hydrolysis directly by chemical quench flow [13], [66], [69], given weak ATP binding to Dbp5 (i.e., saturation requires ~ 20 mM ATP, a small fraction of which will be hydrolyzed even with tens of micromolar Dbp5, making detection by thin-layer chromatography, HPLC, and other conventional means technically challenging). However, isotope exchange experiments and the presence of the lag in P_i_BiP fluorescence time courses provide sufficient evidence to implicate both hydrolysis and P_i_ release as slow and relatively irreversible transitions that limit Dbp5 ATPase cycling in the absence of RNA and other regulatory factors. Current structural and biochemical data support a role for protein regulators (i.e., Gle1-InsP_6_ and Nup159) in facilitating enzyme recycling post-hydrolysis [25], [27], which fits well with the kinetic bottlenecks identified here. Future studies will be required to investigate if these regulators function in this fashion and/or facilitate other transitions in the ATPase cycle that become limiting in the presence of RNA and trans-activating factors.

### Interactions with mant-labeled nucleotides

We found that mant-labeled nucleotides severely perturb the kinetics and thermodynamics of the Dbp5 ATPase cycle. The rate and equilibrium constants are affected by several orders of magnitude and the rate-limiting transitions for intrinsic cycling are also perturbed (Supplementary Information). This is the first example to our knowledge of such dramatic differences in behaviors between native and mant-labeled nucleotides with the DBP family of enzymes. While the origin of this fluorophore effect is still unclear, additional hydrophobic interactions with the mant fluorophore (Fig. 3 and Supplementary Information) are likely to contribute. It is intriguing to speculate if the ability of Dbp5 to discriminate between ATP and ATP-like molecules may serve some additional function *in vivo*, as was recently described for the DBP Ded1 in AMP sensing [70]. Certainly, it demonstrates that fluorophore effects are not uniform even between closely related enzymes and must be checked [33], [35], [48].

### General features of the Dbp5 RNA-stimulated ATPase cycle

Dbp5 steady-state ATPase cycling is accelerated 20-fold by RNA (Table 1). Consequently, the main rate-limiting step in the intrinsic Dbp5 cycle—P_i_ release—must be accelerated by RNA at least 20-fold. Intriguingly, RNA binding (*k*_HT + R_ = 1–5 s^− 1^) becomes partially rate limiting and, together with P_i_ release (*k*_− RPi_ = 1–5 s^− 1^), limits steady-state Dbp5 ATPase cycling in the presence of RNA. This suggests that the interaction time between Dbp5 and RNA is ≥ 0.4 s $\left(\tau_{RNA\;\mathrm{binding}}+\tau_{P_{i}\;\mathrm{release}}=\frac{1}{k_{HT+R}}+\frac{1}{k_{-{RP}_{i}}}\geq \frac{1}{5\;s^{-1}}+\frac{1}{5\;s^{-1}}=0.4\;s\right)$, which may be significant for the role of Dbp5 in mRNA export (see the discussion below) and/or other cellular processes.

### Comparison to other DBPs

The intrinsic Dbp5 ATPase cycle appears very similar to that of other RNA helicases. Like Dbp5, both DbpA and Mss116 display unfavorable hydrolysis and fully or partially rate-limiting P_i_ release in the absence of RNA [13], [14], [15]. In the case of DbpA, hydrolysis is so unfavorable that it is undetectable in the absence of its RNA activator, the peptidyl-transferase center (PTC) of the 23S ribosomal RNA [13], [15], [71]. Although the equilibrium constants for ATP hydrolysis (*K*_H_) and P_i_ release (*K*_Pi_) of intrinsic Dbp5 ATPase activity actually favor progression through the ATPase cycle, the slow rate constants associated with both transitions bottleneck the intrinsic cycle at similar positions (i.e., biochemical states) as with DbpA and Mss116 cycling. In this sense, Dbp5 appears to be typical of the DBP family.

Despite these similarities with other representative DBPs, some unique features of Dbp5 should be noted. First is the extremely weak millimolar ATP binding affinity (~ 4 mM) as compared to sub-millimolar binding affinities for most other studied DBPs [13], [14]. As a result, little Dbp5-ATP is populated under physiological conditions[72], [73]; rather, free Dbp5, Dbp5-ADP-P_i_, and Dbp5-ADP are favored (Fig. 7).

A second feature of the intrinsic ATPase cycle of Dbp5 is the slow reverse hydrolysis (ATP resynthesis) rate constant both in the presence and in the absence of RNA, which maintains Dbp5 in an ADP-P_i_ state when coupled with even slower P_i_ release (Fig. 7). Although accelerated at least 20-fold with RNA, P_i_ release remains at least partially rate limiting, such that Dbp5 significantly populates the ADP-P_i_ state even in the presence of RNA. Formation of such a stabilized biochemical intermediate may promote distinct and/or high-affinity interactions with RNAs important for *in vivo* function, as with other DBPs [10], [13], [14], [15], which may be required for the proposed ribonucleoprotein (RNP) remodeling activity of Dbp5 at NPCs [74], [75], [76].

Alternatively, it has been demonstrated that Dbp5 shuttles through the nucleus [19], interacts with transcriptional machinery [39], and travels with nuclear RNPs from the site of transcription to NPCs [37]. This latter activity may rely on Dbp5 acting as an ATP-dependent RNA clamp and remaining bound to an mRNA, similar to eIFAIII as part of the exon-junction complex[9]. Maintenance of Dbp5 in an RNA-bound state would allow Dbp5 to be loaded on to mRNPs in the nucleus during mRNP biogenesis and subsequently released upon reaching the cytoplasmic side of an NPC where known regulators (i.e., Gle1 and Nup159) are located, resulting in spatial regulation of the Dbp5 ATPase cycle. Within such a model, completion of the ATPase cycle upon exiting the nucleus would facilitate directional mRNP export by initiating disruption of the Dbp5-RNP scaffold and mRNP remodeling. mRNA export times are reported to be in the range of a few hundred milliseconds [77], [78], [79], [80], as such the ability of Dbp5 to remain bound to RNA for ≥ 400 ms is consistent with a clamp model for Dbp5 in mRNA export. To distinguish among these possibilities related to mRNA export and to understand how Dbp5 functions in other cellular processes, it will be critical to continue to investigate how regulators individually, and in combination, alter the Dbp5 ATPase cycle.

## Materials and Methods

### Chemicals

All chemical reagents were of the highest purity commercially available. Millipore MiliQ® water (filtered through a 0.2-μm filter) or Sigma RNase-free water was used in all buffers. ATP (Sigma A7699, ≥ 99% purity assayed by HPLC, ≤ 0.1% inorganic phosphate) and ADP (Roche Molecular Biochemicals) concentrations were determined by absorbance using ε_259_ of 15,400 M^− 1^ cm^− 1^. Polyuridylic acid (polyU RNA) (SC-215733A; Santa Cruz Biotechnology) was dialyzed extensively against distilled deionized water and ethanol precipitated prior to use. RNA concentrations were determined by absorbance using ε_260_ of 9660 M^− 1^ cm^− 1^ and are in units of nucleotides. Mant-labeled nucleotides (2′ and 3′ mixed isomer, mantATP, and mantADP from Invitrogen;mantATP, mant-dATP, and mant-dADP from Jena Biosciences) concentrations were determined by absorbance (ε_255_ = 23,300 M^− 1^ cm^− 1^). One molar equivalent of MgCl_2_ was added to all nucleotides immediately before use.

All assays were performed at 25 ± 0.1 °C in 30 mM Hepes(pH 7.5), 100 mM KCl, 2 mM MgCl_2_, and 2 mM DTT.

### Protein expression, purification, and labeling

N-terminal 6 × His-tagged Dbp5 was expressed and purified from *Escherichia coli* as previously described [81]. The *E*. *coli* phosphate binding protein (P_i_BiP) was purified and labeled with MDCC (7-diethylamino-3-(((2-maleimidyl)ethyl)amino)carbonyl coumarin (Thermo Fisher Scientific Inc.) as previously described [44], [82].

### Protein crystallization

Dbp5 crystal structures used the construct ∆90Dbp5 (Dbp5 residues 91–482) instead of full-length Dbp5 [27], purified as described above. In preparation for crystallization, ∆90Dbp5 was dialyzed into 30 mM Hepes(pH 7.5), 400 mM NaCl, 1 mM DTT, and 0.25 mM InsP_6_ in 10% glycerol as previously described [27]. The N-terminal 6 × His tag was cleaved overnight at 4 °C with tobacco etch virus (TEV) protease in 5 mM DTT. Cleaved ∆90Dbp5 was purified by nickel affinity chromatography with Ni^2 +^-NTA agarose (Qiagen) followed by size-exclusion chromatography over a Superdex 200 (10/300) GL column (GE Healthcare) in 10 mM Hepes(pH 7.5), 100 mM NaCl, 1 mM DTT, and 0.25 mM InsP_6_ in 10% glycerol. Purified ∆90Dbp5 was concentrated after the addition of 5 mM MgCl_2_. The ∆90Dbp5-mantADP·BeF_3_ complex was formed by mixing concentrated ∆90Dbp5 in solution with a 1.2:1 molar ratio of rU_10_ RNA (IDT), incubating 15 min, and then mixing mantADP·BeF_3_ (prepared in a 1:3:15 ratio of ADP:Be:F) to a final concentration of 1 mM, followed by another 15 min of incubation. Crystallization of ∆90Dbp5-mantADP·BeF_3_ was performed by hanging-drop vapor diffusion at 18 °C by mixing 100 nL of the complex at a protein concentration of 16 mg mL^− 1^ with 100 nL of reservoir solution containing 200 mM Mg(NO_3_)_2_ and 18% polyethylene glycol 3350 and incubating 4 days over 100 μL of reservoir solution in a sealed chamber. Crystals were transferred to a cryoprotectant solution containing 18% polyethylene glycol 3350, 9 mM Hepes(pH 7.5), 180 mM Mg(NO_3_)_2_, 4.5% glycerol, 1 mM DTT, 90 mM NaCl, 0.25 mM InsP_6_, 4.5 mM MgCl_2_, and 1 mM mantADP·BeF_3_ (prepared in a 1:3:15 ration of ADP:Be:F) and were flash frozen in liquid nitrogen.

### Crystallographic data collection, structure determination, and refinement

Data were collected at 100 K on the NECAT 24-ID-E beam line of the Advanced Photon Source at the Argonne National Laboratory and processed with HKL [83]. Crystals belonged to the space group *P*2_1_2_1_2_1_. The structure of ∆90Dbp5-mantADP·BeF_3_ was determined by molecular replacement with Phaser using the entire Dbp5 subunit (PDB ID 3PEY) as a search model [84]. The atomic coordinates and temperature factors were refined with REFMAC5, initially with noncrystallographic symmetry restraints applied [85]. The atomic model was improved with cycles of model building with Coot [86] followed by positional refinement. Rigid-body motions were modeled with REFMAC5 in terms of TLS tensors for translation, libration, and screw rotation [87]. Water molecules were added using an automated procedure in Coot and by visual inspection. A final round of refinement was performed with PHENIX [88]. Atomic coordinates and structure factors have been deposited in the Protein Data Bank under accession code 5ELX. See Table S2 for data collection and refinement statistics.

### Steady-state ATPase assays

Time courses of Dbp5 (0.6–1 μM) steady-state ATPase assayed by absorbance change at 340 nm using the NADH-coupled assay were acquired on a Perkin-Elmer Lambda 20 UV–Vis spectrophotometer thermostatted at 25 °C [13], [43], [66]. The [ATP] dependence of the initial steady-state ATPase rate (*v*_0_) was fitted to the Michaelis–Menten equation:

(10) $$v_{0}=\frac{k_{\mathrm{cat}}{\left[\text{H}\right]}_{\text{T}}\left[\text{T}\right]}{\left[\text{T}\right]+K_{\text{M,ATP}}}$$

where *k*_cat_ is the maximal turnover rate of Dbp5 at saturating ATP in units of s^− 1^, [H]_T_ is the total Dbp5 concentration, *K*_M,ATP_ is the Michaelis constant for ATP, and [T] is the total [ATP]. The [ADP] under our conditions of 2 mM ATP is ∼ 7 μM [43]. Steady-state ATPase assays were also performed using ATPγS as a substrate.

RNA-stimulated steady-state ATPase experiments were performed as detailed above, using 100 nM Dbp5 and 20 mM ATP. Data were fitted to the quadratic form of the Briggs–Haldane equation

(11) $$v_{obs}=\left(k_{\mathrm{cat}}-k_{0}\right)\frac{{\left[H\right]}_{T}+{\left[R\right]}_{T}+K_{M\;,\mathrm{RNA}}-\sqrt{{\left({\left[H\right]}_{T}+{\left[R\right]}_{T}+K_{M,RNA}\right)}^{2}-4{\left[H\right]}_{T}{\left[R\right]}_{T}}}{2{\left[H\right]}_{T}}+k_{0}$$

where [R]_T_ is the total RNA concentration in nucleotides [14], [66].

### Transient kinetic assays

Transient kinetic measurements were performed with an Applied Photophysics SX2.0 stopped-flow apparatus thermostatted at 25 ± 0.1 °C [13], [14], [15], [30], [66]. The time courses shown are either of raw, unaveraged traces or the average of 2–3 traces. The concentrations stated are final after mixing. Uncertainties are reported as standard errors in the fits unless stated otherwise. The following minimal reaction scheme with corresponding fundamental rate (*k*) and equilibrium constants (*K*, defined by ratio of corresponding *k* values) was used for analysis and modeling[13], [14], [15], [66]:(Scheme 1)
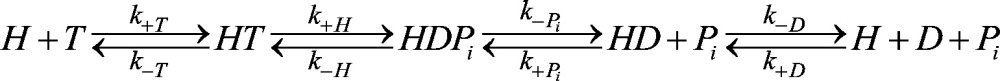
where H is Dbp5, T is ATP, D is ADP, and P_i_ is inorganic phosphate. We assume that product release is sequential with P_i_ preceding ADP. Several processes (e.g., nucleotide binding) are likely to be associated with multiple transitions (e.g., binding followed by an isomerization of the Dbp5 nucleotide complex) but are omitted for simplicity [8], [13], [14], [15], [66].

### Nucleotide binding

Time courses of mant-nucleotide binding were measured by FRET from Dbp5 tryptophans to bound nucleotides. Fluorescence (λ_ex_ = 280 nm) was monitored through a 400-nm-long pass colored filter. Inner filter effects from mant-nucleotide absorption are minimal at the mant-nucleotide concentrations used (< 120 μM). Time courses of association were acquired under both pseudo-first-order and nonpseudo-first-order conditions. Data acquired under pseudo-first-order conditions were fitted to a linear sum of exponentials. Time courses acquired under nonpseudo-first-order conditions were fitted with the following expression[89]:

(12) $$F_{0}+A_{0}\left(1-\frac{r-1}{re^{-k_{0}t}-1}e^{-k_{0}t}\right)+\sum_{i=1}^{n}A_{i}\left(1-e^{-k_{i}t}\right)$$

where *F*_0_ is the signal at time (*t*) = 0, *A*_0_ and *k*_0_ are the signal amplitude and observed rate constant of the first phase, and *r* is an indication of the deviation of the bimolecular binding term [the second term in Eq. (12)] from a single exponential. Under pseudo-first-order conditions, *r* approaches 0 and the second term collapses to an exponential. When pseudo-first-order conditions are not observed, *r* approaches 1, creating significant deviations from a single exponential [89]. The terms *A_i_* and *k_i_* (*i* = 1,…, *n*) are the amplitudes and observed rate constants of additional phases following bimolecular binding, respectively. Irreversible mantADP dissociation was measured by rapidly mixing a pre-equilibrated mixture of 1 μM Dbp5 and 10 μM mantADP with 1 mM ADP and the time courses of fluorescence change were fitted to a sum of exponentials [51], [66].

Unlabeled nucleotide binding was measured by kinetic competition with mant-labeled nucleotides [35], [51]. Time courses of fluorescence change associated with mant-nucleotide binding after rapidly mixing 0.5–0.6 μM Dbp5 with a mixture of 20 or 50 μM mant nucleotide (either mantADP or mantATP) and varying concentrations of unlabeled nucleotide (ATP or ADP) were fitted to a sum of exponentials. The unlabeled nucleotide binding affinity (dissociation equilibrium constant, *K*_d_) was determined from the [unlabeled nucleotide] dependence of the observed mant-nucleotide binding rate constant, as defined by [51], [64]:

(13) $$k_{obs}=\frac{\left(k_{0}-k_{\infty }\right)}{1+\frac{\left[N\right]}{K_{d}}}+k_{\infty }$$

where [N] is unlabeled nucleotide concentration and *k*_0_ is the observed mant-nucleotide rate constant in the absence of competing unlabeled nucleotide ([N] = 0), and *k*_∞_ at saturating [N].

### Transient P_i_ release of intrinsic Dbp5 ATPase

Phosphate release from Dbp5 was assayed from the eightfold increase in fluorescence (λ_ex_ = 436 nm, 463-nm-long pass colored glass emission filter) of the _i_MDCC-labeled PiBiP upon binding phosphate. Contaminating P_i_ was removed from all equipment and buffers with a P_i_ “mop” consisting of 0.5 mM 7-methylguanosine and 0.01 U/mL purine nucleoside phosphorylase (Sigma). Since the process of mop's scavenging free P_i_ in solution is very slow on the seconds timescale and P_i_BiP binds P_i_ much faster (< 100 ms) and extremely tightly, the presence of mop has minimal effect on transient and steady-state P_i_ release measurements, especially at short timescales [62]. Furthermore, 7-methylguanosine (up to 250 μM) does not alter mantATP binding kinetics (data not shown). P_i_BiP fluorescence was converted into [P_i_] following calibration with phosphate standard solutions. Time courses of P_i_ liberation ([P_i_]) measured after rapidly mixing 0.5 μM Dbp5 with an equilibrated mixture of 5 μM P_i_BiP and varying ATP concentrations were fitted to the following expression defining a lag phase followed by a linear phase[13], [14]:

(14) $$\left[P_{i}\right]=\beta \left(\frac{1}{\lambda }\left(e^{-\lambda t}-1\right)+t\right)+P_{i,0}$$

The linear regime represents steady-state ATPase, and its slope (given by β) is the turnover rate. The lag phase is defined by an exponential with observed rate constant λ. The [ATP] dependence of the observed lag phase rate constant was fitted to a hyperbola in the following form[13], [14]:

(15) $$\lambda =\frac{\left(\lambda_{\infty }-\lambda_{0}\right)\left[T\right]}{K_{T}+\left[T\right]}+\lambda_{0}$$

where [T] is the ATP concentration, λ_∞_ is the observed lag phase rate constant at saturating ATP, λ_0_ is the intercept value at zero ATP, and *K*_T_ is the ATP dissociation equilibrium constant.

### RNA-stimulated transient P_i_ release of Dbp5 ATPase

Single-mix experiments were conducted above with the following modifications: 0.4 μM Dbp5 was pre-equilibrated with varying concentrations of RNA and was rapidly mixed with 2.5 mM ATP pre-equilibrated with 5 μM P_i_BiP. P_i_ mop was present in all solutions. Data were analyzed as detailed above.

Dual-mix experiments were conducted as above, with the modification that all solutions contained both 5 μM P_i_BiP and P_i_ mop. Using the dual-mix mode of the SX20 stopped flow, we mixed 5 μM Dbp5 with 4 mM ATP and aged it for 0.05, 6, or 25 s. After aging, we rapidly mixed the Dbp5/ATP solution with 0 or 10 mM RNA (nucleotide). Final concentrations were 2.5 μM Dbp5, 2 mM ATP, 0 or 10 mM RNA, and 5 μM P_i_BiP. Time courses of [P_i_] release in the absence of RNA were fit to a sum of bimolecular binding and a linear function. Time courses of [P_i_] in the presence of RNA were fit to the following sum of bimolecular binding and combined lag/linear phase:

(16) $$\left[P_{i}\right]=F_{0}+A_{0}\left(1-\frac{r-1}{re^{-k_{0}t}-1}e^{-k_{0}t}\right)+\beta \left(\frac{1}{\lambda }\left(e^{-\lambda t}-1\right)+t\right)$$

### Oxygen isotope exchange

ATPase reactions (5–10 μM Dbp5 and 5–10 mM ATP) were conducted in 5–6 mM phosphoenolpyruvate, 50 U mL^− 1^ phosphoenolpyruvate kinase, and 48.5% ^18^O-labeled water. Reactions were run at room temperature for several hours to generate 2–3 mM P_i_, then quenched with an equivalent volume of 1 N HCl. P_i_ was isolated and analyzed by coupled gas chromatography/mass spectrometry (GCMS) [61], [64]. GCMS analysis of P_i_ samples from hydrolysis of unlabeled ATP in ^18^O-enriched water yields the distribution of species with zero to four ^18^O oxygens per P_i_. The *P*_c_ value was not determined by global fitting of this complete distribution because the starting reactions contained variable amounts of unenriched P_i_ that result in an excess of the ^18^O_0_ species. At the low extents of reversal observed with Dbp5, most of the P_i_ contains only one water-derived oxygen with only a small amount of P_i_ that contains two water-derived oxygens. The amount of the species with two water-derived oxygens increases with increasing *P*_c_ value. The ratio of the ^18^O_2_:^18^O_1_ species with 2 *versus* 1 labeled oxygens was therefore used to determine *P*_c_ because this ratio only depends on the enrichment in the water and the *P*_c_ value and is uninfluenced by the amount of contaminating unenriched P_i_.

Oxygen isotope exchange experiments were also performed with RNA-stimulated Dbp5 ATPase activity. Steady-state reactions in the presence of RNA used 2 μM Dbp5, 2.5 mM ATP, and 8 mM polyU RNA. Data were analyzed as above.

### Simulation of transient and steady-state activity

Kinetic simulations by global fitting of both steady-state ATPase time courses acquired with the NADH assay and transient/steady-state P_i_ release acquired by P_i_BiP fluorescence were performed using Scheme 1 and KinTek Global Kinetic Explorer Software (KinTek Corp.) [90], [91]. A rapid (≫*k*_cat_; e.g., ≥ 10 s^− 1^) transition accounting for ATP regeneration from liberated ADP was also included to account for the activity of lactate dehydrogenase and phosphoenolpyruvate kinase in the NADH assay.

### Accession numbers

Coordinates of the crystal structure of Dbp5 bound to RNA and mantADP·BeF_3_ have been deposited in the Protein Data Bank under the accession number 5ELX.

## Figures and Tables

**Table 1:** Rate and equilibrium constants of the Dbp5 ATPase cycle

Parameter	Value	Units	Assay
*Steady*-*state ATPase*			
*k*_cat_	0.043 (± 0.001)	s^− 1^	NADH-linked assay
	0.084 (± 0.002)	s^− 1^	P_i_BiP
*K*_M,ATP_	1.7 (± 0.2)	mM	NADH-linked assay
	1.3 (± 0.1)	mM	P_i_BiP

*Steady*-*state ATPase of ATPγS*			
*k*_cat_	0.036 (± 0.001)	s^− 1^	NADH-linked assay
*K*_M,ATPγS_	0.98 (± 0.06)	mM	NADH-linked assay

*ATP and mantATP binding*			
*k*_+ mT1_	1.63 (± 0.04)	μM^− 1^ s^− 1^	mantATP
*k*_− mT1_	11.9 (± 0.7)	s^− 1^	mantATP
*K*_mT1_	7.3 (± 0.4)	μM	*k*_−_/*k*_+_
*k*_+ mT2_	0.4 (± 0.6)	s^− 1^	mantATP
*k*_− mT2_	1.9 (± 0.4)	s^− 1^	mantATP
*K*_mT2_	5 (± 7)		*k*_−_/*k*_+_
*k*_mT3,obs_	0.01 (± 1 × 10^− 4^)	s^− 1^	mantATP
*K*_mT,overall_	1 (± 0.3)	μM	Total fluorescence amplitude
*K*_T_	6.4 (± 0.7)	mM	mantATP competition
	3.0 (± 0.4)	mM	mantADP competition
	4 (± 2)	mM	P_i_BiP

*ATP hydrolysis*			
*k*_+ H_	2.2 (± 0.4)	s^− 1^	P_i_BiP
*k*_− H_	2 × 10^− 4^ (± 5 × 10^− 5^)	s^− 1^	P_i_BiP and isotope exchange
*K*_H_	1 x 10^− 4^ (± 3 × 10^− 5^)		*k*_−_/*k*_+_

*Phosphate release*			
*k*_− Pi_	0.02 (± 0.1)	s^− 1^	P_i_BiP
*k*_+ Pi_	< 2 × 10^− 6^	μM^− 1^ s^− 1^	*k*_− Pi_/*K*_Pi_
*K*_Pi_	10	mM	Steady-state P_i_ inhibition

*ADP and mantADP binding*			
*K*_mD0_	102 (± 21)	μM	mantADP
*k*_+ mD1_	98 (± 15)	s^− 1^	mantADP
*k*_− mD1_	0.2 (± 0.5)	s^− 1^	mantADP
	2.611 (± 0.003)	s^− 1^	Irreversible mantADP dissociation
*K*_mD1_	0.002 (± 0.005)		*k*_−_/*k*_+_ mantADP
*k*_mD2,obs_	~ 0.7 (± 0.1)	s^− 1^	mantADP
*K*_D_	0.36 (± 0.05)	mM	mantADP competition

*RNA*-*stimulated steady*-*state ATPase activity*			
*k*_cat_	0.92 (± 0.08)	s^− 1^	NADH-linked assay
*K*_M,RNA_	3.4 (± 0.8)	mM	NADH-linked assay

*Oxygen isotope exchange*			
*P*_c_	0.0416 (± 0.0025)		GCMS

**Table 2:** Rate and equilibrium constants determined from global fitting

Parameter	Value	Units	Assay
*Steady*-*state ATPase activity*			
*k*_cat_	0.04 (± 0.0001)	s^− 1^	Eq. (8)
*K*_M,ATP_	1.4 (± 1.1)	mM	Eq. (9)

*ATP binding*			
*k*_+ T_	0.92 (± 0.03)	μM^− 1^ s^− 1^	KinTek steady-state simulation
*k*_− T_	3670	s^− 1^	KinTek steady-state simulation
*K*_T_	4	mM	Constrained from competition assay

*ATP hydrolysis*			
*k*_+ H_	0.16 (± 5 × 10^− 4^)	s^− 1^	KinTek steady-state simulation
	0.72 (± 0.01)	s^− 1^	KinTek P_i_BiP simulation
*k*_− H_	6 × 10^− 4^ (± 8 × 10^− 7^)	s^− 1^	KinTek steady-state simulation
	0.38 (± 0.05)	s^− 1^	KinTek P_i_BiP simulation
*K*_H_	0.004 (± 5 × 10^− 4^)		KinTek steady-state simulation
	0.53 (± 0.07)		KinTek P_i_BiP simulation

*Phosphate release*			
*k*_− Pi_	0.062 (constrained)	s^− 1^	KinTek steady-state simulation
	0.04 (± 0.02)	s^− 1^	KinTek P_i_BiP simulation
*k*_+ Pi_	< 1 × 10^− 6^ (constrained)	s^− 1^	KinTek steady-state simulation
	< 1 × 10^− 6^	s^− 1^	KinTek P_i_BiP simulation
*K*_Pi_	> 1 × 10^− 4^	μM	KinTek steady-state simulation
	> 1 × 10^− 4^	μM	KinTek P_i_BiP simulation

*ADP release*			
*k*_+ D_	0.2	μM^− 1^ s^− 1^	KinTek steady-state simulation
*k*_− D_	64 (± 16)	s^− 1^	KinTek steady-state simulation
*K*_D_	0.31	mM	Constrained from competition assay
