# Supplementary material for: Pi Release Limits the Intrinsic and RNA-Stimulated ATPase Cycles of DEAD-Box Protein 5 (Dbp5)
Source: J Mol Biol. 2016 Jan 29;428(2Part B):492–508. doi: 10.1016/j.jmb.2015.12.018 (PMC4744555; doi:10.1016/j.jmb.2015.12.018)
Supplement: Supplementary file 1 — Supplementary material [file mmc1.docx]

**SUPPLEMENTARY INFORMATION**

***Materials and Methods***

*Transient kinetics of mantATP hydrolysis*

Transient kinetics of mantATP hydrolysis were performed on a KinTek chemical quench flow, model RQF-3, thermostatted at 25 ± 0.1 °C. Samples were quenched with 1 M formic acid. mantATP and mantADP were separated by high performance liquid chromatography (HPLC) using a Targa C18 column (Higgins Analytical, Inc.) in a gradient of buffer A (8 mM phosphate buffer, pH 6, 20% methanol) with 0-20% buffer B (acetonitrile).^1^ Nucleotides were detected by absorbance at 259 and 350 nm.

***Results***

*Transient kinetic analysis of the Dbp5 mantATPase cycle*

*mantATP binds Dbp5 tightly and in multiple steps*

Because some biochemical transitions of the ATPase cycle are associated with conformational changes in the active site, the FRET signal from mant-nucleotide binding likely reflect steps other than the initial binding phase.^2-6^ Time courses of mantATP binding to Dbp5 are well fitted by three exponentials (Fig. 2 and Table 1). The fastest observed rate constant (*k*_mT1,obs_) depends linearly on the [mantATP] (Fig. 2) and reflects bimolecular binding (e.g. formation of Dbp5-mantATP; Scheme 1). The second fastest phase (*k*_mT2,obs_) depends hyperbolically on the [mantATP] and possibly represents an isomerization after binding and preceding hydrolysis (omitted from Scheme 1; discussed below under *mantATPase cycling is limited by hydrolysis or P*_i_ *release*) or hydrolysis itself (see below). Globally fitting the [nucleotide]-dependence of the two fast observed rate constants to the following equation

$\begin{matrix} k_{fast,slow}=\frac{1}{2}\left(k_{+mT1}[mT]+k_{-mT1}+k_{+mT2}+k_{-mT2}\right. \\ \left.\pm\sqrt{\left(k_{+mT1}[mT]+k_{-mT1}+k_{+mT2}+k_{-mT2}\right)^{2}-4\left(k_{-mT2}k_{-mT1}+k_{-mT2}k_{+mT1}[mT]+k_{+mT2}k_{+mT1}[mT]\right)}\right) \end{matrix}$ Equation 17.

yields the mantATP association and dissociation rate constants from the slope and intercept of the best fit of *k*_1_ (*k*_+mT1_ = 1.6 µM^-1^ s^-1^ and *k*_−mT1_ = 11.9 s^-1^, Table 1) and the rate constants of the second phase described by *k*_2_ (*k*_+mT2_ = 0.4 s^-1^ and *k*_−mT2_ = 1.9 s^-1^). The third slower phase (*k*_mT3,obs_) depends weakly on [mantATP] over the concentration range examined (0 - 100 µM) and appears to reflect an additional downstream isomerization at ~0.01 s^-1^, which may reflect mantATP hydrolysis (discussed below under *mantATPase cycling is limited by hydrolysis or P*_i_ *release*) or P*_i_* release.

*Multiphasic mantATP binding traces are not due to dye isomerization*

mant-labeled nucleotide exists in solution as a mixture of 2' and 3' mant-linkages, which sometimes display different kinetics and thermodynamics on interaction with nucleotide-binding proteins.^7-9^ To evaluate if the multiple phases do not reflect differential binding of mixed 2' and 3' mantATP isomers, nucleotide binding experiments as described in the main text (Fig. 2) were carried out with mant-2' deoxyATP, which mimics the 3' mantATP isomer (Table S1). Time courses of mant-2' deoxyATP binding to Dbp5 are well fitted by four exponentials. The 3'- (represented by mant-2' deoxy) and 2'/3' mixed isomer second order association rate constants differ only slightly (*k*_+mT1_ = 1.9 µM s^-1^ for mixed isomers; *k*_+mdT1_ = 1.5 µM s^-1^ for 2' deoxy isomer). In contrast, the dissociation rate constants are markedly different (*k*_−mT1_ = 8 s^-1^ and *k*_−mdT1_ = 224 s^-1^). Rapid dissociation of the 3' mant isomer is absent from time courses of mixed isomer binding, suggesting that the 3' mant isomer contributes minimally to the mixed isomer binding signal and that it is dominated by the 2' mant isomer. The Dbp5/RNA/mantADP•BeF_3_ crystal structure (Figs. 3, S4, S5) contains 2' mant nucleotide in its active site (even though both 2' and 3' were present during crystallization), suggesting that Dbp5 preferentially selects 2' mant over 3' mant-labeled

We also assayed binding of mantATPγS, a common “non”- or slowly-hydrolyzable ATP analog (Table S1). Steady-state ATPase assays demonstrate that Dbp5 can utilize both ATP and ATPγS as substrates with near-equal efficiency (Table 1). However, compared to mantATP, time courses of mantATPγS binding contain an additional phase immediately following bimolecular binding, with *k*_+mγS2_ of 1.7 s^-1^ and a *k*_−mγS2_ of 11.9 s^-1^, and a slightly decreased amplitude in the final phase (Table 1 and Table S1). Interestingly, although the second phase of mantATP binding occurs almost ten-fold more slowly than that of mantATPγS, the second phase of mant-2' deoxyATP binding (*k*_mdT2,obs_ = 13 s^-1^) occurs on a very similar timescale. Based on this observation, it is possible that both mantATPγS and mant-2' deoxyATP, as unconventional substrates, may favor an alternate, weakly- or non-hydrolysis competent Dbp5 conformation. Time courses of the non-hydrolyzable ATP analog, mantAMPPNP, binding are also multiphasic, but the initial binding affinity is weaker than that of mantATP and the rates do not appear to directly correspond to any of the observed rates from mantATP binding (Table S1).

*2' and 3' mantADP bind Dbp5 tightly*

Time courses of fluorescence change after mixing Dbp5 and mantADP are multiphasic and best described by two exponentials (Fig. 2, Table 1). The fastest observed phase is hyperbolic with [mantADP] and represents a biochemical transition following bimolecular binding, which occurs within the dead time of the measurement (≤ 3 ms).^2, 10-12^ Fitting the fastest observed phase to a rectangular hyperbola gives *K*_mD0_ = 102 µM for initial mantADP binding (*k*_mD0_). The apparent overall affinity for the first observed phase and the fast bimolecular binding phase (*K*_mD,overall_ = *K*_mD0_*K*_mD1_/(*K*_mD0_+*K*_mD1_)) is significantly tighter than *K*_mD0_ due to the relative irreversibility of the transition described by *k*_−mD1_. The second observed biochemical transition (third overall) in mantADP binding occurs at 0.7 s^-1^ and depends weakly on [mantADP].

To obtain a better estimate of *k*_−mD1_, irreversible mantADP dissociation was measured by mixing an equilibrated Dbp5-mantADP mixture with 1 mM ADP (i.e. unlabeled competitor). Time courses of fluorescence change are well fitted by a single exponential with an observed rate constant of 2.6 s^-1^. This value is ten-fold faster than *k*_−mD1_ estimated from the extrapolation of *k*_mD1,obs_ to the y-intercept (Table 1). The latter is subject to large uncertainty.

Time courses of mant-2' deoxyADP (mimicking 3' mantADP) binding are also best fitted by three exponentials. The fastest observed rate constant associated with most of the observed signal depends linearly on the [mant-2' deoxyADP] and presumably reflects bimolecular binding, yielding a second-order association rate constant of 2.9 µM^-1^ s^-1^ from the slope of the best linear fit and an apparent dissociation rate constant of 197 s^-1^ from the intercept (Table S1). As noted for mantATP above, the rapid dissociation of 3' mantADP is not observed in time courses of mantADP mixed isomer binding, suggesting again that adenosine nucleotides with the 2' mant linkage bind preferentially over those with the 3' linkage.

*mantATP is hydrolyzed by Dbp5*

Since mant nucleotide induces very different nucleotide binding behavior from Dbp5 and because a hydrolysis step was not definitively distinguished with slowly- and non-hydrolyzable mantATP analogs, it is not clear if Dbp5 recognizes mantATP as a substrate for hydrolysis and subsequent ATPase cycle transitions. We therefore evaluated if Dbp5 is capable of using mantATP as a nucleotide substrate for ATPase cycling. Attempts to perform steady-state mantATPase experiments using the NADH coupled assay system were unsuccessful as the coupled assay failed to reliably detect mantADP product. In contrast, P*_i_* release assayed by P*_i_*BiP fluorescence provided a reliable measure of steady state mantATPase cycling. P*_i_*BiP fluorescence also provides information on the kinetics of hydrolysis and subsequent P*_i_* release with mantATP as a substrate.

Time courses of P*_i_* release from Dbp5 with mantATP substrate, in contrast to those with unlabeled ATP (Fig. 4), do not display a [nucleotide]-dependent lag phase (Fig. S1). The maximal steady-state cycling rates (*k*_cat_) with ATP and mantATP are comparable and occur at ~0.05 s^-1^, suggesting that the slowest phase (~0.01s^-1^) in mantATP binding reflects the rate-limiting step. However, the *K*_M_ for mantATP (*K*_M,mT_) is 17 µM, orders of magnitude tighter than that of unlabeled ATP (Table S1).

Time courses of P*_i_*BiP fluorescence provide clear evidence that Dbp5 is capable of utilizing mantATP as an ATPase substrate. mantATPase activity is fundamentally different from unlabeled ATPase activity, given the lack of a [mantATP]-dependent lag phase in P*_i_* release time courses as well as the values of the individual substrate *K*_M_ and *K*_d_ values.

Assuming a similar minimal ATPase scheme applies to mantATPase activity, the slow *k_cat_* of 0.054 s^-1^ suggests that similar to unlabeled ATP, mantATP and mantADP binding to Dbp5 are rapid and that hydrolysis and/or P*_i_* release must be rate limiting instead. The lack of a detectable lag in P*_i_* release time courses favors a mechanism with a single rate limiting step − either hydrolysis is slow and rate-limiting and followed by rapid phosphate release, or hydrolysis is rapid followed by slow, rate-limiting P*_i_* release. Alternatively, an additional isomerization(s) distinct from hydrolysis or P*_i_* release (not included in Scheme 1) may occur slowly and limit overall cycling with mantATP.

*mantATPase cycling is limited by hydrolysis or P*_i_ *release*

To determine the timescale of hydrolysis, mantATPase reactions were performed on a quench-flow with Dbp5 (12 µM) in slight excess over mantATP (11 µM). mantATP reactants and mantADP products were separated and quantitated by HPLC. Time courses of mantADP production deviate from a single exponential because the reaction is carried out under non-pseudo first order conditions (i.e. [substrate] ≈ [enzyme]), and binding is explicitly dependent on the concentration of both reactants. In addition, depletion of both reactants gradually slows binding during the time course of association. This appears as an apparent slow phase in the time course, allowing it to be well fitted by a double exponential. The fast observed rate constant (*k*_obs_ = 0.02 s^-1^) of the double exponential fit completed in the earliest part of the time course when both [substrate] and [enzyme] are not significantly depleted represents bound mantATP hydrolysis. The second, slow observed rate constant (*k*_obs_ ~0.001 s^-1^; Fig. S2) arises from enzyme/substrate depletion.^13^ The slow timescales of observed hydrolysis (~0.02 s^-1^) are in the range expected for a transition limiting mantATPase cycling (~0.05 s^-1^).

To determine the corresponding timescales of nucleotide binding and P*_i_* release, mantATP FRET signal and P*_i_*BiP fluorescence was monitored under similar single turnover conditions (5 µM P*_i_*BiP, and Dbp5 at 6.75 µM in excess over mantATP at 6 µM). The time courses of mantATP FRET signal contain three phases with observed rate constants of 11 s^-1^, 0.03 s^-1^, and 0.006 s^-1^ (Fig. S3). The fastest phase occurs on timescales expected for bimolecular binding under multiple turnover conditions (Fig 2, Table 1). As the *K*_mT1_ is 7 µM (Table 1), neither the population of free enzyme nor the population of free substrate are significantly depleted by bimolecular binding alone. The binding phase under our single turnover conditions should thus mirror those of multiple turnover conditions. In addition, the relative rapidity of this phase permits its decoupling from downstream, slower processes. Consequently, the first downstream step (at 0.03 s^-1^) is not initially limited by insufficient substrate binding and can be used to estimate the rough timescales of hydrolysis and P*_i_* release, while the slowest phase (0.006 s^-1^) should be an additional phase introduced by the significant depletion of both reactants, as seen in quench flow/HPLC measurements. mantADP product remains bound under these reactant concentrations and conditions, so the equilibrium fluorescence does not decay. P*_i_*BiP fluorescence, like quench flow/HPLC traces, features two phases with observed rate constants of 0.02 s^-1^ and 0.004 s^-1^. As before, the slow phase is assumed to represent substrate depletion, while the fast observed rate constant reflects P*_i_* release occurring at 0.02 s^-1^.

The observed behaviors from chemical quench flow, mant FRET signal and P*_i_*BiP fluorescence under single turnover condition are consistent with two possible kinetic schemes, each defined by a single rate-limiting transition. The observation that P*_i_* release (Fig. S3) occurs on the same time scale as the chemical cleavage of bound mantATP measured by quench flow (Fig. S2) is consistent with a mechanism in which hydrolysis is rate limiting (~0.02 s^-1^) and subsequent P*_i_* release is rapid. Chemical cleavage at 0.02 s^-1^ is also consistent with the third phase (*k*_mT3,obs_) observed in mantATP binding under pseudo first conditions (Fig. 2, Table 1). In this scheme, the second phase observed in mantATP binding (*k*_mT2,obs_) under pseudo first conditions (Fig. 2, Table 1) is more rapid than hydrolysis (0.02 s^-1^) and must therefore represent a conformational change after binding and preceding hydrolysis. However, the data do not eliminate an alternative scheme with rapid, but unfavorable hydrolysis and slow and rate-limiting P*_i_* release. Time courses of mantATP binding data under pseudo first order conditions reveal an isomerization (*k*_mT2,obs_ ~2 s^-1^) after mantATP binding and before the rate limiting step (~0.01 s^-1^; Fig. 2, Table 1). The isomerization could reflect hydrolysis (with *k*_obs_ equal to sum of forward and reverse rate constants). If so, P*_i_* release would be rate limiting at 0.01 s^-1^ (*k*_mT3,obs_). In this case, the unfavorable *K*_mT2_ (*k*_−_/*k*_+_ = 5; Table 2 of main text) and *k*_mT2_ value (2 s^-1^) that is much faster than P*_i_* release (0.01 s^-1^) precludes a strong ADP-P*_i_* burst in hydrolysis, as observed.

Both schemes adequately explain the data without additional information. However, it is notable that the proposed kinetic behaviors differ fundamentally from that of unlabeled ATP, which features both irreversible hydrolysis and rate-limiting P*_i_* release. We therefore conclude that the mantATP consumption follows a different catalytic cycle than unlabeled ATP, and does not directly translate to the equilibrium or kinetic behavior of unlabeled substrate.

**Table S1. Rate and equilibrium constants of Dbp5 mantATPase activity**

| **Parameter** | **Value** | **Units** | **Assay** |
| --- | --- | --- | --- |
|  |  |  |  |
| *Steady-state ATPase activity* | | | |
| *k*_cat,mT_ | 0.054 (±0.002) | s^-1^ | P*_i_*BiP |
| *K*_M,mT_ | 17.2 (±0.9) | µM | P*_i_*BiP |
|  |  |  |  |
| *mant-2' deoxyATP binding* | | | |
| *k*_+mdT1_ | 1.5 (±0.5) | µM^-1^ s^-1^ | mant-2' deoxyATP |
| *k*_−mdT1_ | 224 (±28) | s^-1^ | mant-2' deoxyATP |
| *K*_mdT1_ | 68 (±10) | µM | *k*_−_/*k*_+_ |
| *k*_mdT2,obs_ | 13 (±2) | s^-1^ | mant-2' deoxyATP |
| *k*_mdT3,obs_ | 1 (±0.5) | s^-1^ | mant-2' deoxyATP |
| *k*_mdT4,obs_ | 0.004 (±0.001) | s^-1^ | mant-2' deoxyATP |
|  |  |  |  |
| *mantAMPPNP binding* | | | |
| *k*_+mPNP1_ | 0.52 (±0.03) | µM^-1^ s^-1^ | mantAMPPNP |
| *k*_-mPNP1_ | 64 (±2) | s^-1^ | mantAMPPNP |
| *K*_PNP1_ | 123 (±8) | µM | *k*_−_/*k*_+_ |
| *k*_mPNP2,obs_ | 4 (±5) | s^-1^ | mantAMPPNP |
| *k*_mPNP3,obs_ | 0.02 | s^-1^ | mantAMPPNP |
|  |  |  |  |
| *mATPγS binding* | | | |
| *k*_+mγS1_ | 2.9 (±0.1) | µM^-1^ s^-1^ | mantATPγS |
| *k*_-mγS1_ | 24 (±2) | s^-1^ | mantATPγS |
| *K*_mγS1_ | 8.3 (±0.7) | µM | *k*_−_/*k*_+_ |
| *k*_+mγS2_ | 1.7 (±1) | s^-1^ | mantATPγS |
| *k*_-mγS2_ | 11.8 (±0.6) | s^-1^ | mantATPγS |
| *k*_mγS3,obs_ | 1.3 (±0.1) | s^-1^ | mantATPγS |
| *k*_mγS4,obs_ | 0.003 (±0.003) | s^-1^ | mantATPγS |
|  |  |  |  |
| *mantATP hydrolysis* | | | |
| *k*_H,obs_ | 0.023 (±0.004) | s^-1^ | STO*^1^* quench flow/HPLC |
|  |  |  |  |
| *Phosphate release* | | | |
| *k*_−P_*_i_*_,obs_ | 0.02 (±3 × 10^-5^) | s^-1^ | STO*^1^* P*_i_*BiP assay |
|  |  |  |  |
| *mant-2' deoxyADP binding* | | | |
| *k*_+mdT1_ | 2.9 (±0.3) | µM^-1^ s^-1^ | mant-2' deoxyADP |
| *k*_−mdT1_ | 197 (±20) | s^-1^ | mant-2' deoxyADP |
| *K*_mdT1_ | 68 (±10) | µM | *k*_−_/*k*_+_ |
| *k*_mdT2,obs_ | 9 (±8) | s^-1^ | mant-2' deoxyADP |
| *k*_mdT3,obs_ | 0.2 (±0.1) | s^-1^ | mant-2' deoxyADP |

Abbreviations: *1*. STO, single turnover

**Table S2. Crystallographic data collection and refinement statistics**

|  | ∆90Dbp5-mantADP•BeF_3_ | |
| --- | --- | --- |
| **Data collection** |  | |
| Space group | *P 2_1_ 2_1_ 2_1_* | |
| Cell dimensions |  | |
| *a*, *b*, *c* (Å) | 42.1, 91.8, 104.5 | |
| α, β, γ (º) | 90, 90, 90 | |
| Wavelength (Å) | 0.97918 | |
| Resolution (Å)^a^ | 45.4 - 1.81 (1.88 - 1.81) | |
| Unique reflections | 37,475 | |
| *R*_merge_ (%)^a,b^ | 8.2 (51.6) | |
| *I* / σ*I*^a^ | 10.6 (1.78) | |
| Completeness (%)^a^ | 99 (100) | |
| Redundancy^a^ | 2.0 (2.0) | |
|  |  | |
| **Refinement** |  | |
| Resolution (Å) | 45.4 -1.81 | |
| No. reflections (working set) | 37,471 | |
| No. reflections (test set) | 1,846 | |
| *R*_work_ / *R*_free_^c^ (%) | 17.5 / 20.8 | |
| Averaged B factor (Å^2^)^d^ | 17.3 | |
| No. atoms | 3,627 | |
| Protein | 3,247 | |
| Water | 325 | |
| Ligand | 55 | |
| R.M.S^e^ deviations |  | |
| Bond lengths (Å) | 0.005 | |
| Bond angles (º) | 1.06 | |
| **Ramachandran analysis** | |  |
| Preferred regions (%) | 98.0 | |
| Allowed regions (%) | 1.7 | |
| Disallowed regions (%) | 0.0 | |
| Synchrotron Beamline | APS NE-CAT 24ID-C | |

^a^Highest resolution shell is shown in parentheses.

^b^*R*_merge_ = Σ_hkl_Σ_i_ |I_hkl,i_ – < I>_hkl_| / Σ_hkl_Σ_i_|I_hkl,i_|, where I_hkl_ is the intensity of a reflection and <I>_hkl_ is the average of all observations of the reflection.

^c^*R_free_*, *R_work_* with 10% of F_obs_ sequestered before refinement.

^d^Residual B-factors after TLS refinement. See PDB entry for TLS refinement parameters.

^e^R.M.S., root mean square.

Figure S1. Transient and steady-state P*_i_* release during Dbp5 mantATPase activity. (a) Time courses of P*_i_* release assayed by fluorescence change after mixing 0.5 µM Dbp5 with 5 µM P*_i_*BiP and (lower to upper curves) 0, 5, 15, or 30 µM mantATP. Bold lines through the data represent the best fits to linear functions with slopes corresponding to the steady-state ATPase rates. (b) [mantATP]-dependence of the Dbp5 steady state mantATPase rate. The continuous line through the data represents the best fit to a rectangular hyperbola, yielding the maximum velocity per enzyme (*k*_cat,mT_) from the amplitude and the *K*_M,mT_ from the [mantATP] at half-maximum velocity (Table S1). Uncertainty bars represent standard errors in the fits and are within the data points.

Figure S2. Time course of mantADP formation acquired under single turnover conditions. Dbp5 (12 µM) was rapidly mixed with mantATP (11 µM) and quenched with formic acid at various times. mantATP substrate and mantADP product were separated by HPLC and quantitated. The solid line through the data points represents the best fit to a linear sum of two exponentials. The fast observed rate constant of mantADP formation occurs at 0.02 s^-1^ and the slower, apparent relaxation can be defined by a 0.001 s^-1^ observed rate constant. The observed burst phase amplitude under these non-saturating mantATP concentrations is 0.25 (3 µM mantADP/12 µM Dbp5).

Figure S3. Time courses of FRET signal change and P*_i_* release acquired under single turnover conditions. (a) Time course of mant-nucleotide fluorescence (FRET) change after mixing 6.75 µM Dbp5 with 5 µM P*_i_*BiP and 6 µM mantATP. The smooth line through the data represents the best fit to a linear sum of three exponentials with rate constants of 11 s^-1^, 0.03 s^-1^, and 0.006 s^-1^. The inset shows data over a shorter time scale. (b) Comparison of time courses of FRET (from Panel (a)) and P*_i_*BiP (5 µM) signal change after mixing 6.75 µM Dbp5 with 6 µM mantATP. The line through the P*_i_*BiP data represents the best fit to a linear sum of two exponentials with rate constants of 0.02 s^-1^ and 0.004 s^-1^. Data is plotted with offset and on a log scale for clarity.


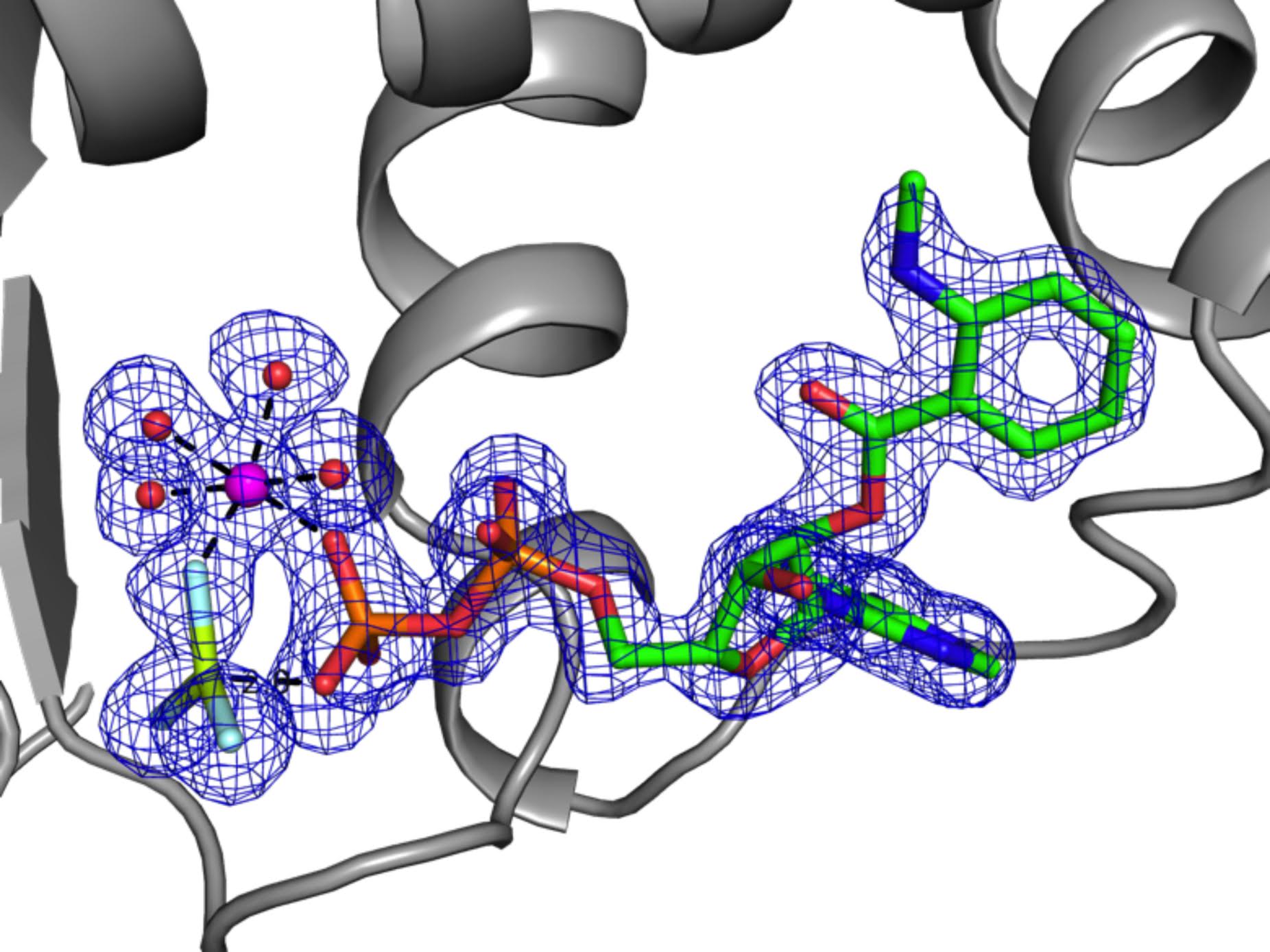


Figure S4. *F_o_* - *F_c_* omit electron density map (magenta) of ∆90Dbp5-mantADP•BeF_3_ calculated before adding the nucleotide ligand, bound waters, and Mg^2+^ ion to the atomic model. A contour level of σ = 3 was used for map calculation.


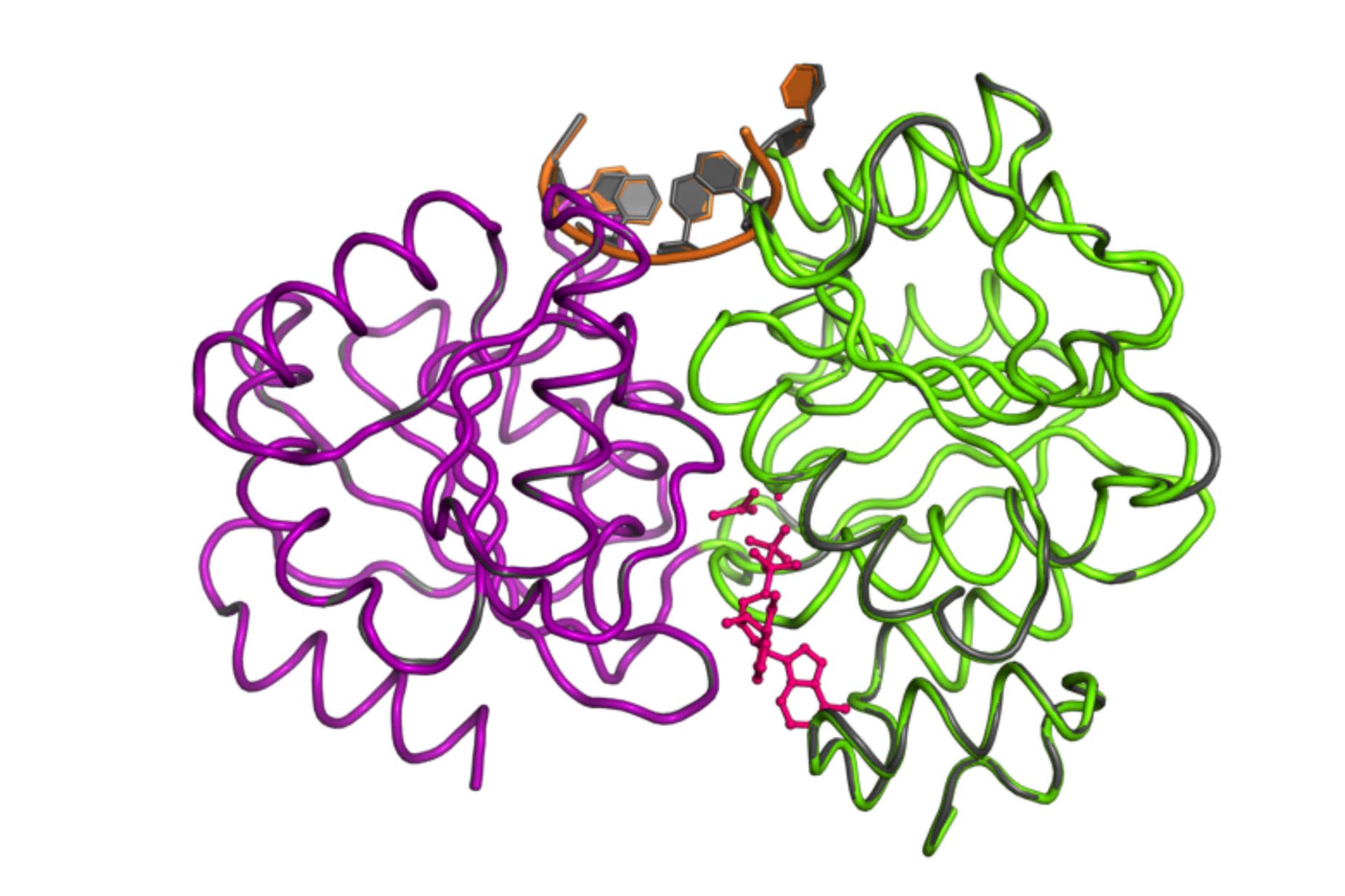


Figure S5. The crystal structure of ∆90Dbp5-mantADP•BeF_3_ superimposed on the ADP•BeF_3_ –bound structure. The ADP•BeF_3_-bound structure is in grey (PDB ID **3PEY**). The mant-ADP•BeF_3_-bound structure is colored as follows: N-terminal domain (green), C-terminal domain (magenta), RNA (orange), mantADP•BeF_3_ (pink). The root mean square deviation (rmsd) of the atomic coordinates in the two structures is 0.111 Å.

**SUPPLEMENTARY MATERIAL REFERENCES**

1. Jameson D. M., Eccleston J. F. (1997). [18] Fluorescent nucleotide analogs: Synthesis and applications. Methods Enzymol. Volume 278, 363-390.

2. Bradley M. J., De La Cruz E. M. Analyzing ATP Utilization by DEAD-Box RNA Helicases Using Kinetic and Equilibrium Methods. (2012). In Methods Enzymol. (Eckhard J, editor. eds), pp. 29-63, Academic Press.

3. Moore K. J. M., Lohman T. M. (1994). Kinetic mechanism of adenine nucleotide binding to and hydrolysis by the Escherichia coli Rep monomer. 1. Use of fluorescent nucleotide analogs. Biochemistry. 33, 14550-14564.

4. Henn A., Cao W., Hackney D. D., De La Cruz E. M. (2008). The ATPase Cycle Mechanism of the DEAD-box rRNA Helicase, DbpA. J. Mol. Biol. 377, 193-205.

5. Cao W., Coman M. M., Ding S., Henn A., Middleton E. R., Bradley M. J., et al. (2011). Mechanism of Mss116 ATPase Reveals Functional Diversity of DEAD-Box Proteins. J. Mol. Biol. 409, 399-414.

6. Toseland C. P., Webb M. R. (2013). ATPase Mechanism of the 5′-3′ DNA Helicase, RecD2: Evidence for a Pre-Hydrolysis Conformation Change. J. Biol. Chem. 288, 25183-25193.

7. Cheng J.-Q., Jiang W., Hackney D. D. (1998). Interaction of Mant-Adenosine Nucleotides and Magnesium with Kinesin. Biochemistry. 37, 5288-5295.

8. Eccleston J. F., Moore K. J. M., Brownbridge G. G., Webb M. R., Lowe P. N. (1991). Fluorescence approaches to the study of the p21ras GTPase mechanism. Biochem. Soc. Trans. 19, 432-437.

9. Hiratsuka T. (1983). New ribose-modified fluorescent analogs of adenine and guanine nucleotides available as subtrates for various enzymes. Biochim. Biophys. Acta. 742, 496-508.

10. Henn A., Bradley M. J., De La Cruz E. M. (2012). ATP Utilization and RNA Conformational Rearrangement by DEAD-Box Proteins. Annu. Rev. Biophys. 41, 247-267.

11. Pollard T. D., De La Cruz E. M. (2013). Take advantage of time in your experiments: a guide to simple, informative kinetics assays. Mol. Biol. Cell. 24, 1103-1110.

12. De La Cruz E. M., Michael Ostap E. Chapter 6 Kinetic and Equilibrium Analysis of the Myosin ATPase. (2009). In Methods Enzymol. eds), pp. 157-192, Academic Press.

13. Cao W., De La Cruz E. M. (2013). Quantitative full time course analysis of nonlinear enzyme cycling kinetics. Sci. Rep. 3, 2658.
